# Cationic Cascade Strategy for the Synthesis of Dihydrobenzofuran and Isochromane Scaffolds

**DOI:** 10.1021/acs.orglett.5c04731

**Published:** 2026-01-06

**Authors:** Patrycia K. Zybura, Kyla J. Grant, Alison J. Frontier

**Affiliations:** Department of Chemistry, 6927 University of Rochester, Rochester, New York 14627-0216, United States

## Abstract

Oxygen heterocycles are valued for their prevalence in bioactive molecules and their metabolic stability. We report a Brønsted-acid-promoted, oxygen-interrupted *halo*-Prins/*halo*-Nazarov cascade that enables the stereospecific construction of dihydrobenzofuran and isochromane scaffolds from simple enyne and carbonyl precursors. This approach unites *de novo* heterocycle construction with late-stage functionalization, providing access to complex oxygen heterocycles of relevance to medicinal chemistry.

## Graphical abstract

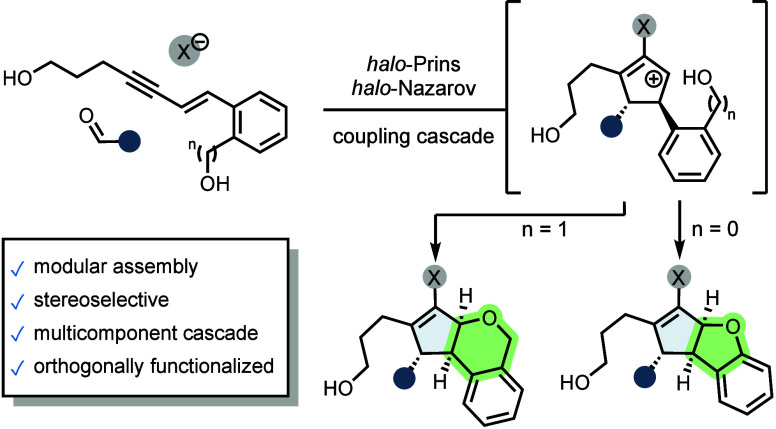

Heterocycles comprise nearly one-third of all known organic compounds and play a central role in pharmaceutical research and drug development due to their prevalence in bioactive molecules.^1^ Synthetic approaches generally fall into two categories: functionalization of pre-existing heterocycles and *de novo* construction from acyclic precursors, with the latter often favored for densely substituted or unconventional motifs.

Oxygen-containing heterocycles represent one of the most common heterocyclic classes, second only to their nitrogen analogues. Among them, benzofurans and isochromanes are notable for their presence in bioactive molecules.^2,3^ Previous *de novo* syntheses have relied on transition metal catalysis and the pre-installation of directing groups or coupling partners to direct reactivity (Scheme 1A).^4,5^

**Scheme 1:**
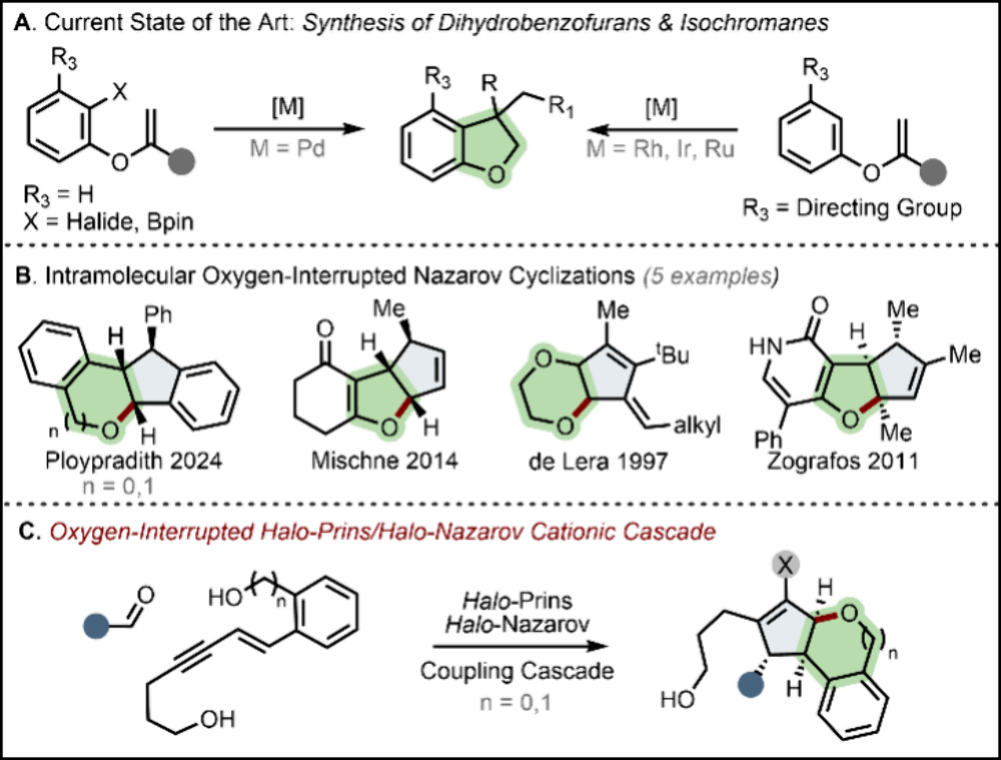
Cascade Reactions for the Synthesis of Dihydrobenzofuran and Isochromane Scaffolds

Cationic cascades are powerful tools for the rapid generation of molecular complexity. Among these, interrupted Nazarov cyclizations can combine stereospecific electrocyclization with intramolecular trapping, generating two rings when the cyclopentenyl cation intermediate is captured with a tethered nucleophile. The majority of these cascades conclude with the formation of a C–C bond, to generate complex fused carbocyclic systems.^6^ In contrast, Nazarov cationic cascades that terminate with C–heteroatom bond formation, delivering cyclopentane-fused nitrogen or oxygen heterocycles, are relatively rare.^7,8^ Indeed, only five tandem cyclizations that feature terminal capture with oxygen have been disclosed, enabling the synthesis of four types of oxygen-containing ring systems (Scheme 1B).^9^

In this letter, we present a concise approach in which simple precursors can be combined to generate a *halo*-allyl cation, which undergoes capture by a pendent oxygen nucleophile (Scheme 1C). This strategy forms three unique bonds in one pot (C–Br, C–O, and C–C) and three contiguous stereocenters, enabling the stereospecific construction of functionally dense *O*-heterocycles.

The investigation of the O-interrupted *halo*-Prins/*halo*-Nazarov cascade began with the synthesis of enyne **3** via a three-step sequence: Ramirez olefination, Hirao reduction, and Sonogashira coupling (see the Supporting Information for more information). Next, we tried using different protecting groups on phenol **3** to aid in the suppression of possible undesired side reactions in the first *halo*-Prins step. However, we found that unprotected phenol outperforms methoxymethyl ether (MOM), *tert*-butyldimethylsilyl, and methyl-protected phenol derivatives in the *halo*-Prins and *halo*-Nazarov steps.

Therein, we continued reaction optimization with unprotected enyne **3** and benzaldehyde, focusing on the effects of different halide sources, acids, and solvents (Table 1). We found that tetrabutylammonium bromide (TBABr) (entry 1) outperforms both tetrabutylammonium iodide (TBAI) (entry 2) and tetrabutylammonium chloride (TBACl) (entry 3). Next, different acids and acid loadings were explored. Using 1.25 equiv of Tf_2_NH (entry 1) works well in the reaction cascade, generating 82% of **5a**. However, using TfOH instead leads to a decrease in the yield of **5a** (entry 6). Increasing the acid loading decreases the yield with both acids (entries 4 and 5). Finally, we began investigating different solvents for this reaction. We found that chlorinated solvents work the best overall, with CH_2_Cl_2_ outperforming CHCl_3_ (entries 1 and 8, respectively). Non-chlorinated solvents are less efficient (see the Supporting Information for more information). The reaction yield also drops when the overall temperature is increased (see the Supporting Information for more information). While the *halo*-Prins and *halo*-Nazarov steps can be carried out individually, the overall yield of the sequence is lower.

**Table 1:**
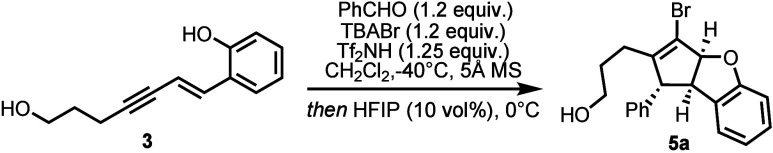
Optimization of Reaction Conditions for the Cyclization Cascade

entry	deviations from optimized conditions	yield (%)
1	none	82
2	TBAI instead of TBABr	78
3	TBACl instead of TBABr	23
4	Tf_2_NH (2 equiv)	42
5	TfOH (2 equiv)	50
6	TfOH (1.2 equiv)	34
7	TMSBr	23
8	CHCl_3_ instead of CH_2_Cl_2_	51
9	HFIP (2 vol %)	60

With the optimized conditions established, we next explored the substrate scope by examining a range of aldehyde reactants. Particular emphasis was placed on selecting aldehydes bearing diverse functional groups or exhibiting a C­(sp^3^)-rich character, both to assess functional group tolerance and to generate a library of C­(sp^3^)-rich products containing synthetically versatile handles, as seen in Scheme 2.

**Scheme 2:**
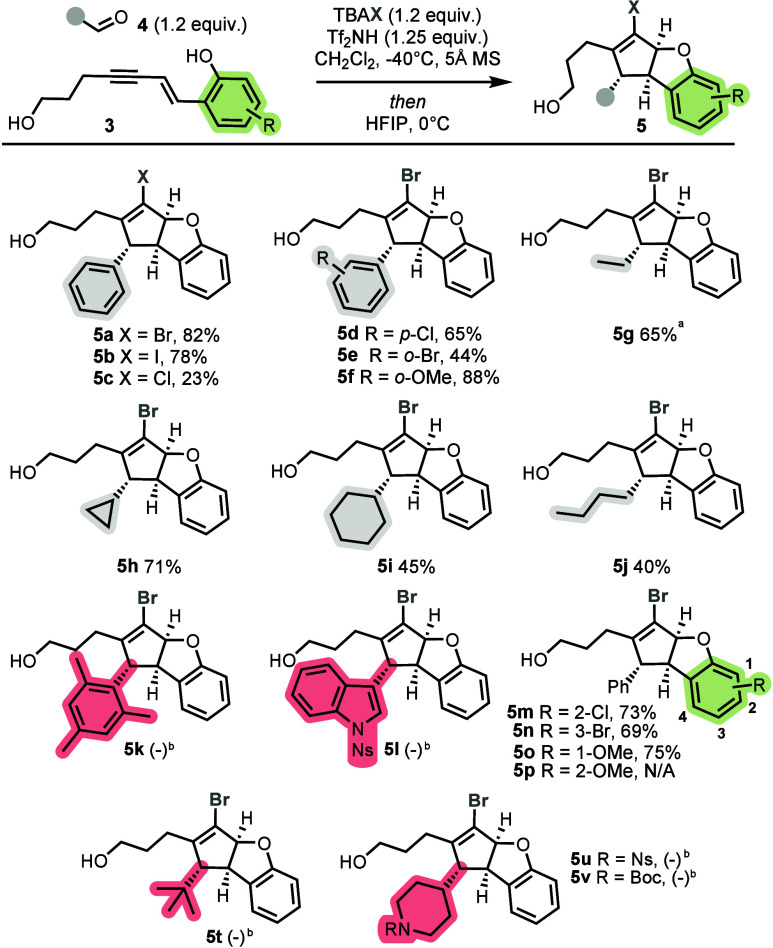
Exploration of Carbonyl Coupling Partners and Phenol Substituent Effects in the Formation of Dihydrobenzofurans

a A total of 2.0 equiv of aldehyde used.
b No product was observed.

Dihydrobenzofuran products bearing functionalized arene rings are formed in generally good yields (44–88%, **5d**–**5f**). We next evaluated aliphatic aldehydes. In general, aliphatic aldehydes yield lower amounts of product compared to their aromatic counterparts, although the yields remained synthetically useful (40–71%, **5g**–**5j**). Notably, employing cyclopropylcarboxaldehyde delivered the desired product **5h** in 71% yield, with the cyclopropane ring remaining intact. Increasing the alkyl chain length leads to a diminished efficiency (**5j**). Use of more sterically challenging aldehydes, such as mesitaldehyde and pivaldehyde, led to no observed product (**5k** and **5t**). Unfortunately, this approach does not tolerate some N-protected heteroaromatic and heteroaliphatic aldehydes. Nosyl-protected indole-3-carbaldehyde (**5l**) and nosyl-protected or Boc-protected piperidine derivatives (**5u** and **5v**) fail to yield any of the desired product under the standard conditions.

We next investigated the effect of varying substitution patterns on the phenol ring of enyne **3**, to understand their influence on the second step of the Nazarov cyclization, specifically, the intramolecular capture of the *halo*-allyl cation by the phenolic hydroxyl group. We hypothesized that different functional groups would modulate the reactivity and efficiency of this key cyclization step.

Substituents at the C2 and C3 positions had modest effects on the reaction outcome. The introduction of halogens at these positions (**5m** and **5n**) results in only a slight decrease in the yield relative to the unsubstituted phenol (82% vs 73 and 69%, respectively). A methoxy group at C1 (**5o**) similarly led to a minor reduction in the yield. In contrast, the placement of a methoxy group at the C2 position (**5p**) completely suppresses product formation, resulting in complex mixtures. We propose that strong electron donation from the C2 methoxy group leads to overactivation of the alkyne–alkene π system, promoting uncontrolled reactivity and diversion from the desired pathway. To test the scalability of this approach, the synthesis of **5a** was carried out on a 1.0 mmol scale, affording the desired product without any change in the yield (82%).

Next, to evaluate the generality of this strategy, we examined whether increasing the tether length of the oxygen nucleophile could enable access to larger ring systems. Specifically, we hypothesized that extending the phenol nucleophile by one carbon to a benzyl alcohol (enyne **6**) could give us access to isochromane scaffolds.

We then reviewed a representative scope of the carbonyl partners (Scheme 3). Aromatic aldehydes engage readily under the reaction conditions and afford the desired products in good yields (70%, **7a**). We then turned our attention to heterocyclic aldehydes, evaluating their performance under the same, unoptimized conditions previously applied to the dihydrobenzofuran series. Both thiophene-2-carboxaldehyde and furfural aldehyde delivered the corresponding products in moderate to good yields (**7b** and **7c**, 65–73%). Finally, we explored a limited set of aliphatic aldehydes and ketones. In general, these substrates give improved outcomes in the isochromane series compared to the dihydrobenzofuran analogues (82% for **7d** vs 45% for **5i**), likely due to the varying nucleophilicity/p*K*_a_ of benzyl versus phenol alcohol.^10^ Cyclohexanone can be used to generate quaternary carbon-containing isochromanes in good yields (**7e**, 81%).

**Scheme 3:**
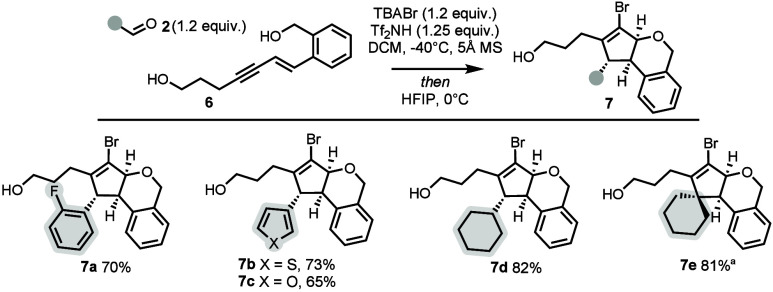
Scope of Carbonyls for Isochromane Scaffolds

a A total of 2.0 equiv of **2** used.

To further demonstrate the synthetic versatility of our product scaffolds, we explored a series of derivatization reactions (Scheme 4). Lithium–halogen exchange of the vinyl bromide moiety using *n*-BuLi results in efficient debromination to generate trisubstituted alkene (**5q**). Oxidation of the pendent alcohol using periodic acid and catalytic chromium oxide proceeds cleanly to furnish the corresponding carboxylic acid (**5s**). Suzuki–Miyaura cross-coupling with phenylboronic acid affords the corresponding styrene derivative (**5r**) in 85% yield.

**Scheme 4:**
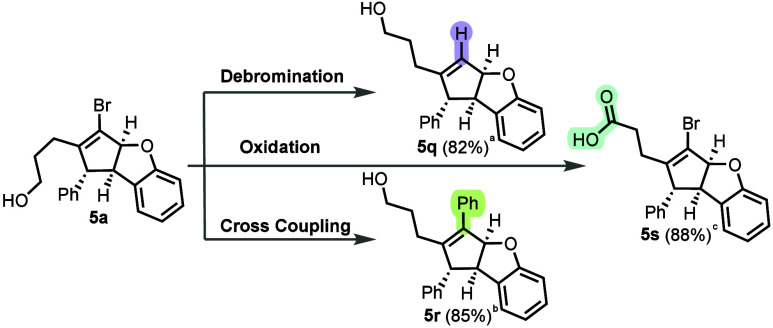
Product Diversification of Dihydrobenzofurans

a Reaction conditions: *n*-BuLi (4.0 equiv) and THF at −78 °C.
b Reaction conditions: PhB­(OH)_2_ (1.5 equiv), Pd­(PPh_3_)_4_ (2.5 mol %), Na_2_CO_3_ (2.0 equiv), and toluene/EtOH/H_2_O at 80 °C.
c Reaction conditions: PCC (2 mol %), H_5_IO_6_ (2.2 equiv), and MeCN at 24 °C.

The proposed mechanism is outlined in Scheme 5. The cascade is initiated by Brønsted acid activation of carbonyl **2**, promoting intermolecular condensation with the pendent alcohol to generate oxocarbenium intermediate **1a**. This species undergoes alkynyl *halo*-Prins cyclization to afford adduct **1b**. Subsequent addition of hexafluoroisopropanol (HFIP) facilitates ionization of the labile C–O bond, forming *halo*-pentadienyl cation **1c**. This intermediate undergoes a 4π conrotatory electrocyclization, establishing two stereocenters (stage 1) and generating *halo*-allyl cation **1d**. In the final step, this cation is trapped by the oxygen atom (stage 2) in a diastereoselective manner, furnishing the desired O-heterocyclic product, **3**.

**Scheme 5:**
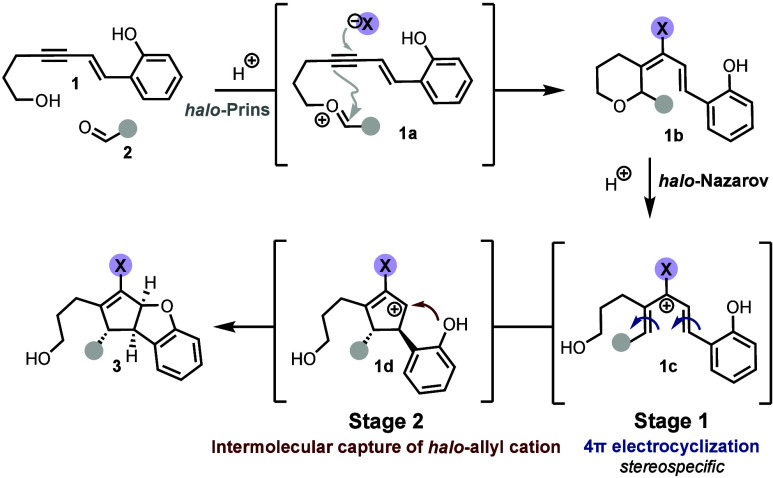
Proposed Mechanism of the *halo*-Prins/*halo*-Nazarov Cascade

In summary, we have developed a Brønsted-acid-promoted, oxygen-interrupted *halo*-Prins/*halo*-Nazarov cascade that enables the stereospecific synthesis of structurally diverse oxygen-containing heterocycles from simple enyne and carbonyl precursors. This method exploits a multicomponent approach that merges two powerful strategies in heterocycle synthesis: *de novo* construction from acyclic building blocks and late-stage functionalization via strategically positioned reactive handles. These features, combined with the privileged nature of oxygen heterocycles in medicinal chemistry, underscore the utility of this strategy for accessing complex, stereochemically rich dihydrobenzofuran and isochromane scaffolds relevant to drug discovery.

## Supplementary Material



## Data Availability

The data underlying this study are openly available in figshare at 10.60593/ur.d.30849737.

## Notes

Representative experimental procedures, detailed experimental procedures, solvent screening, and NMR spectra (PDF)
