# Supplementary material for: Cationic Cascade Strategy for the Synthesis of Dihydrobenzofuran and Isochromane Scaffolds
Source: Org Lett. 2026 Jan 6;28(2):658–62. doi: 10.1021/acs.orglett.5c04731 (PMC12814527; doi:10.1021/acs.orglett.5c04731)
Supplement: Supplementary file 1 [file ol5c04731_si_001.pdf]

Supporting Information

Cationic Cascade Strategy for the Synthesis of Dihydrobenzofuran and Isochromane Scaffolds

Patrycia K. Zybura, Kyla J. Grant, Alison J. Frontier*

Department of Chemistry, University of Rochester,
Rochester, NY, 14627-0216, USA

alison.frontier@rochester.edu*

Table of Contents:

Materials and Methods…………………………………………………………S2

List of Abbreviations……………………………………………………………...S3

Experimental Details …………………………………………………………......S4

Preparation of Enynes………………………………………………………......S4

Halo-Prins/*Halo*-Nazarov Reactions of Phenol Enynes……………………….......S10

Solvent Screening Table………….…………………………………………...S19

Halo-Prins/*Halo*-Nazarov Cascade of Benzyl Enynes……………………….......S20

Derivatization of Dihydrobenzofuran products…………………………………S24

References…………………………………………………………………...…….S27

NMR Spectra……………………………………………………………………S2

Materials & Methods

All reactions were carried out under an argon atmosphere using standard Schlenk techniques unless otherwise noted. Solvents (THF stabilized with BHT, DCM without stabilizers, DMF; Fisher Scientific) (HFIP; Oakwood Chemicals) were dried over activated 4 Å molecular sieves and used under ambient atmosphere. Molecular sieves (4 Å, powdered 5 Å; Aldrich) were stored at 120 °C and further dried by microwave irradiation in 1 min intervals until condensation no longer appeared on the flask neck (after every interval, any condensation that did appear was wiped with a kim wipe). Heating was discontinued if sieves glowed red, as this caused degradation. Activated sieves were cooled under high vacuum, flushed with argon, and added to solvents. Heavy-walled screw-cap tubes (Chemglass, CG-1880) were employed for reactions requiring elevated temperatures/pressures.

Flash column chromatography was performed on silica gel (60 Å, 230–400 mesh; EM Science) using ACS-grade solvents. Reaction progress was monitored by TLC on silica gel 60 F254 plates (EMD), visualized by UV irradiation and p-anisaldehyde staining. Preparative TLC was conducted on the same plates.

Deuterated solvents were obtained from Cambridge Isotope Laboratories. $CDCl_3$ with TMS was stored over flame-dried K_2CO_3 to suppress acid impurities. For NMR samples, ~0.7 mL of solvent was filtered through a cotton plug.

Commercial bis(trifluoromethanesulfonyl)imide (Tf_2NH; TCI America) was dispensed into flame-dried, argon-filled vials (~1 g per vial) and stored at –20 °C. Due to its hygroscopicity, Tf_2NH was added to reactions by weighing by difference into the reaction flask (dipping the spatula into the reaction mixture to ensure all was added, and getting a new spatula every time more had to be added).

NMR Spectroscopy

^{1}H NMR spectra were recorded at 400 MHz and ^{13}C NMR spectra at 101 MHz (Bruker Avance instruments). Chemical shifts (δ) are reported in ppm relative to residual solvent peaks (δ = 7.26 for $CHCl_3$ for ^{1}H, δ = 77.0 for $CHCl_3$ for ^{13}C, or built-in MNova values for other solvents). Data are presented as chemical shift, multiplicity (s = singlet, d = doublet, t = triplet, q = quartet, p = pentet, m = multiplet, and combinations thereof), coupling constants (J, Hz), and integration.

List of Abbreviations

DMF = Dimethylformamide

THF = Tetrahydrofuran

MeCN = Acetonitrile

DCM = Dichloromethane

LAH = Lithium Aluminum Hydride

HFIP = Hexafluoroisopropanol

TBABr = Tetrabutylammonium Bromide

TBACl = Tetrabutylammonium Chloride

TBAI = Tetrabutylammonium Iodide

TLC = Thin Layer Chromatography

FCC = Flash Column Chromatography

BHT = Butylated hydroxytoluene

PCC = Pyridinium chlorochromate

PhMe = Toluene

TFE = Trifluoroethanol

TMS = Trimethylsilane

Experimental Details

Preparation of Phenol Enynes

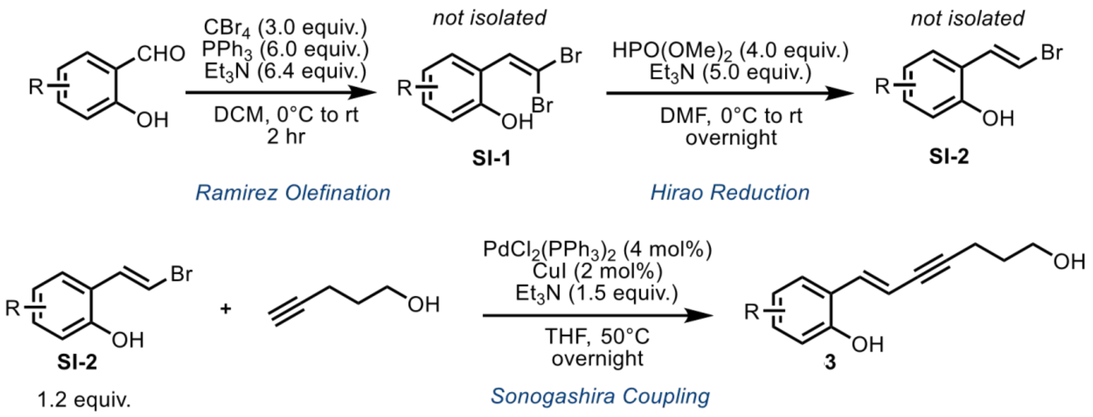

Ramirez Olefination: Compounds **SI-1** were prepared according to the literature procedure using the corresponding substituted salicylaldehyde starting materials.[1] Vinyl dibromides were used as crude directly in the next step.

Hirao Reduction; General Procedure: To a 250 mL round-bottom flask, equipped with a stir bar, **SI-1** (30 mmol, 1.0 equiv.) was added, followed by triethylamine (5.0 equiv.) and DMF (1.0 M). The flask was closed using a yellow cap and the mixture was stirred and cooled to 0°C using an ice bath. Once cooled, the flask was opened and dimethyl phosphite was added (4.0 equiv.) and the flask was recapped. After 10 minutes, the flask was taken out of the ice bath and left to stir at room temperature overnight. Starting material and products have a similar Rf (~0.82 in 30% ethyl acetate/hexanes for all **SI-2**), but stain differently using *p*-anisaldehyde. *Quench*: The reaction mixture was diluted with water and the mixture was transferred to a separatory funnel using diethyl ether. The aqueous layer was extracted with diethyl ether. The organic layer was washed with 2M aq. HCl (pH level was checked using a universal pH strip to confirm an excess of HCl), then sat. aq. sodium bicarbonate, then brine. The organic layer was dried by the addition of excess anhydrous magnesium sulfate (filtered over a fritted funnel under vacuum, collected solvents were removed by vacuum distillation using a rotary evaporator to obtain crude **SI-2** to be used in the next step). *Note: These products are not bench stable and change color if left at room temperature or if exposed to excess light. Store in the freezer covered with tin foil.*

Sonogashira Coupling; General Procedure: To a pressure flask, **SI-2** (30 mmol, 1.2 equiv.), triethylamine (3.0 equiv.), and THF (0.1 M) were added. A septum pierced with a long needle and a bleed needle was fit onto the neck of the flask, with an argon balloon connected to the long needle to degas the reaction mixture. After 30 minutes of continuous degassing, the degassing needle was removed, $PdCl_2(PPh_3)_2$ (2 mol%), CuI (4 mol%), and 4-pentyn-1-ol (1.0 equiv.) were added sequentially. The pressure vial cap was screwed on tightly, and the mixture was placed in a preheated 50°C oil bath. The reaction was left to stir overnight at 50°C. *Quench*: Pressure vial was taken out of the oil bath and left to cool to room temperature. Once cooled, the reaction was diluted with ethyl acetate and vacuum filtered through celite into a separatory funnel. Celite was washed with excess ethyl acetate. The mixture was washed with 2M aq. HCl (the pH was checked with a universal pH strip to confirm that there was an excess of acid). The aqueous layer was extracted 3x with ethyl acetate. Then the combined organic layers were washed with water, sat. aq. sodium bicarbonate, and brine. The organic layer was dried by the addition of excess anhydrous magnesium sulfate. The suspension was filtered over a fritted funnel under vacuum. Solvent was removed by vacuum distillation using a rotary evaporator. The crude was purified by flash column chromatography (SiO_2, toluene was used first to remove a yellow band of palladium impurities, then 20% ethyl acetate/hexanes to elute the desired product) to obtain **3.**

Note: Sonogashira coupling workup of products **3b** *and* **3c** *requires thorough extraction of the aqueous layer with ethyl acetate till the desired product is not present in the aqueous layer (checked via TLC). Furthermore,* **3b** *and* **3c** *noticeably darken when kept at room temperature for longer than 2 hours. Storing them in the freezer when not in active use is recommended to prevent degradation of the material.*

Compound 3a

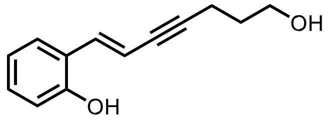

Compound **3a** was isolated as a white solid after column chromatography (toluene to 30% ethyl acetate/hexanes) in 85% yield (3.43 g).

M.P. 87-88°C

This compound has been made before in our group, see the reference for full characterization data. [3]

Compound 3b

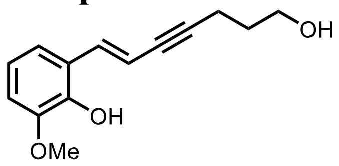

Compound **3b** was isolated as a white solid after column chromatography (toluene to 30% ethyl acetate/hexanes) in 85% yield (3.94 g).

M.P. 64-65°C

^{1}H NMR (400 MHz, $CDCl_3$) δ 7.12 (d, J = 16.4 Hz, 1H), 6.96 (dt, J = 7.3, 1.5 Hz, 1H), 6.83 – 6.73 (m, 2H), 6.30 – 6.21 (m, 1H), 5.95 (s, 1H), 3.88 (d, J = 1.2 Hz, 3H), 3.79 (d, J = 5.6 Hz, 2H), 2.49 (td, J = 6.8, 3.7 Hz, 2H), 1.92 – 1.75 (m, 2H). **^{13}C NMR** ($CDCl_3$, 101 MHz): δ (ppm) 146.7, 143.5, 135.3, 122.9, 119.6, 118.99, 110.0, 109.6, 81.1, 62.0, 56.2, 31.4, 16.3. **HRMS** (ESI-Orbitrap) m/z calc'd for $C_{14}H_{18}O_3$ [M+H]+ : 235.1329 found: 235.1331.

Compound 3c

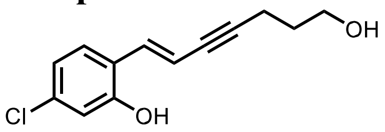

Compound **3c** was isolated as bright yellow oil after column chromatography (toluene to 30% ethyl acetate/hexanes) in 75% yield (3.54 g).

^{1}H NMR (400 MHz, $CDCl_3$) δ 7.30 (s, 1H), 7.10 – 7.04 (m, 1H), 7.00 (d, *J* = 16.2 Hz, 1H), 6.68 (dd, *J* = 8.5, 1.4 Hz, 1H), 6.18 (dq, *J* = 16.5, 2.0 Hz, 1H), 3.79 (t, *J* = 6.1 Hz, 2H), 2.50 (dd, *J* = 7.9, 5.8 Hz, 2H), 1.87 – 1.76 (m, 2H), 1.55 (s, 1H). **^{13}C NMR**($CDCl_3$, 101 MHz): δ (ppm) 151.47, 145.56, 133.79, 128.89, 126.60, 126.38, 125.42, 117.32, 111.31, 80.45, 61.93, 31.38, 16.32. **HRMS** (ESI-Orbitrap) m/z calc'd for $C_{13}H_{13}ClO_2$ [M+H]+ : 237.0677, found: 237.0695.

Compound 3d

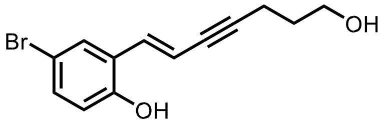

Compound **3d** was isolated as a yellow oil after column chromatography (toluene to 30% ethyl acetate/hexanes) in 68% yield (3.82 g).

^{1}H NMR (400 MHz, $CDCl_3$) δ 7.43 (d, *J* = 2.0 Hz, 1H), 7.19 (dt, *J* = 8.6, 1.9 Hz, 1H), 6.99 (d, *J* = 16.3 Hz, 1H), 6.64 (dd, *J* = 8.6, 1.4 Hz, 1H), 6.21 – 6.11 (m, 1H), 3.79 (t, *J* = 6.1 Hz, 2H), 2.50 (td, *J* = 7.0, 3.7 Hz, 1H), 1.82 (dd, *J* = 7.3, 5.8 Hz, 2H). **^{13}C NMR** ($CDCl_3$, 101 MHz): δ (ppm) 152.3, 133.8, 131.8, 129.5, 125.9, 117.8, 113.2, 111.3, 92.6, 80.5, 61.9, 31.4, 16.3. **HRMS** (ESI-Orbitrap) m/z calc'd for $C_{13}H_{13}BrO_2$ [M+H]+ : 281.0177, found: 281.0175.

Preparation of Benzyl Enynes

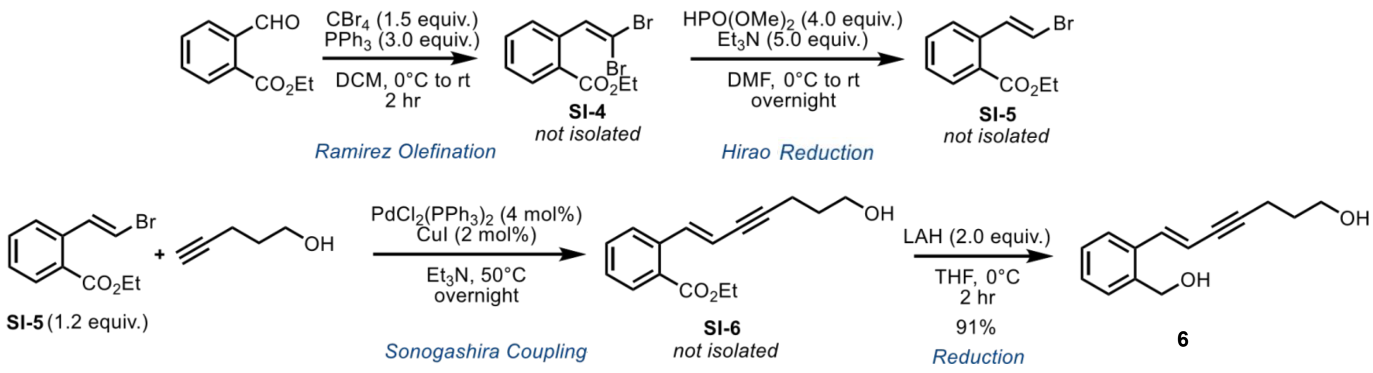

Ramirez Olefination: Compound **SI-4** was prepared according to the literature procedure using ethyl 2-formylbenzoate as the starting aldehyde.[1] Crude **SI-4** was used directly in the next step.

Hirao Reduction: To a 250 mL round-bottom flask, equipped with a stir bar, crude **SI-4** (30 mmol, 1.0 equiv.) was added, followed by triethylamine (5.0 equiv.) and DMF (1 M). The flask was capped with a yellow cap, and the mixture was stirred and cooled to 0°C in an ice bath. Once cooled, the flask was opened and dimethyl phosphite was added (4.0 equiv.) and the flask was recapped. After 10 minutes, the flask was taken out of the ice bath and left to stir at room temperature overnight. Starting material and product have a similar Rf (~0.80 in 30% ethyl acetate/hexanes), but **SI-5** stains purple in *p*-anisaldehyde. *Quench*: The reaction mixture was diluted with water, and the mixture was transferred to a separatory funnel using diethyl ether. The aqueous layer was extracted with diethyl ether. The organic layer was washed with 2M aq. HCl (pH level was checked using a universal pH strip to confirm an excess of HCl), then sat. aq. sodium bicarbonate, then brine. The organic layer was dried by addition of excess anhydrous magnesium sulfate (filtered over a fritted funnel under vacuum, collected solvents were removed by vacuum distillation using a rotary evaporator to obtain crude **SI-5** to be used in the next step). *These products are not bench stable and change color if left at room temperature or if exposed to excess light. Store in the freezer covered with tin foil.*

Sonogashira Coupling: To a 250 mL pressure flask, **SI-5** (30 mmol, 1.2 equiv.), and triethylamine (0.1 M) was added. A septum pierced with a long needle and a bleed needle was fit onto the flask, with an Argon balloon connected to the long needle to degas the reaction mixture. After 30 minutes of continuous degassing, the degassing

needle was removed, $PdCl_2(PPh_3)_2$ (2 mol%), CuI (4 mol%), and 4-pentyn-1-ol (1.0 equiv.) were added sequentially. The pressure vial cap was screwed on tightly, and the mixture was placed in a preheated 50°C oil bath. The reaction was left to stir overnight at 50°C. (Rf of product **SI-6** is ~0.3 in 30% ethyl acetate/hexanes and stains a medium brown using *p*-anisaldehyde) *Quench*: Pressure vial was taken out of oil bath and left to cool to room temperature. Once cooled, the reaction was diluted with ethyl acetate and vacuum filtered through celite into a separatory funnel. Celite was washed with excess ethyl acetate. The mixture was washed with 2M aq. HCl (the pH was checked with a universal pH strip to confirm that there was an excess of acid), water, sat. aq. sodium bicarbonate, and brine. The organic layer was dried by addition of excess anhydrous magnesium sulfate. The suspension was then filtered over a fritted funnel under vacuum, collected solvents were removed by vacuum distillation using a rotary evaporator to obtain **SI-6**. Crude **SI-6** was used directly in the next step without purification.

Reduction: To a 50 mL round-bottom a stir bar was added. The flask was flame-dried under vacuum, left to cool to room temperature under vacuum, then left under argon. Lithium aluminum hydride (LAH, 2.0 equiv.) was added to the flask under argon. The flask containing the solid LAH was cooled to 0°C using an ice water bath. Once cooled, THF (5 mL) was added. The suspension was left to stir rapidly at 0°C. **SI-6** (0.5 mmol, 1.0 equiv.) was added dropwise to the suspension. The reaction was left to stir at 0°C for 2 hours. (Rf of product **6** is ~0.15 and stains a dark chocolate brown using *p*-anisaldehyde) *Quench*: Mixture was quenched using the Fieser workup, following known protocol.[2] The crude was purified by flash column chromatography (SiO_2, 30% ethyl acetate/hexanes to elute product) to obtain **6** as an off-white solid in 91% yield.

M.P. 56°C
^{1}H NMR (400 MHz, $CDCl_3$) δ 7.51 – 7.44 (m, 1H), 7.40 – 7.32 (m, 1H), 7.27 (q, J = 4.9 Hz, 2H), 7.20 (d, J = 16.1 Hz, 1H), 6.10 (dq, J = 16.2, 2.0 Hz, 1H), 4.74 (d, J = 5.5 Hz, 2H), 3.78 (q, J = 5.8 Hz, 2H), 2.50 (t, J = 6.9 Hz, 2H), 1.82 (p, J = 6.6 Hz, 3H). **^{13}C NMR** ($CDCl_3$, 101 MHz): δ (ppm) 137.6, 137.1, 135.4, 128.7, 128.5, 128.4, 125.5, 110.9, 92.2, 80.5, 63.4, 61.9, 31.4, 16.3. **HRMS** (ESI-Orbitrap) m/z calc'd for $C_{14}H_{16}O_2$ [M+H]+ : 217.1223, found: 217.1215.

General Reaction Scheme for *Halo*-Prins/*Halo*-Nazarov Cascade of Phenol Enynes

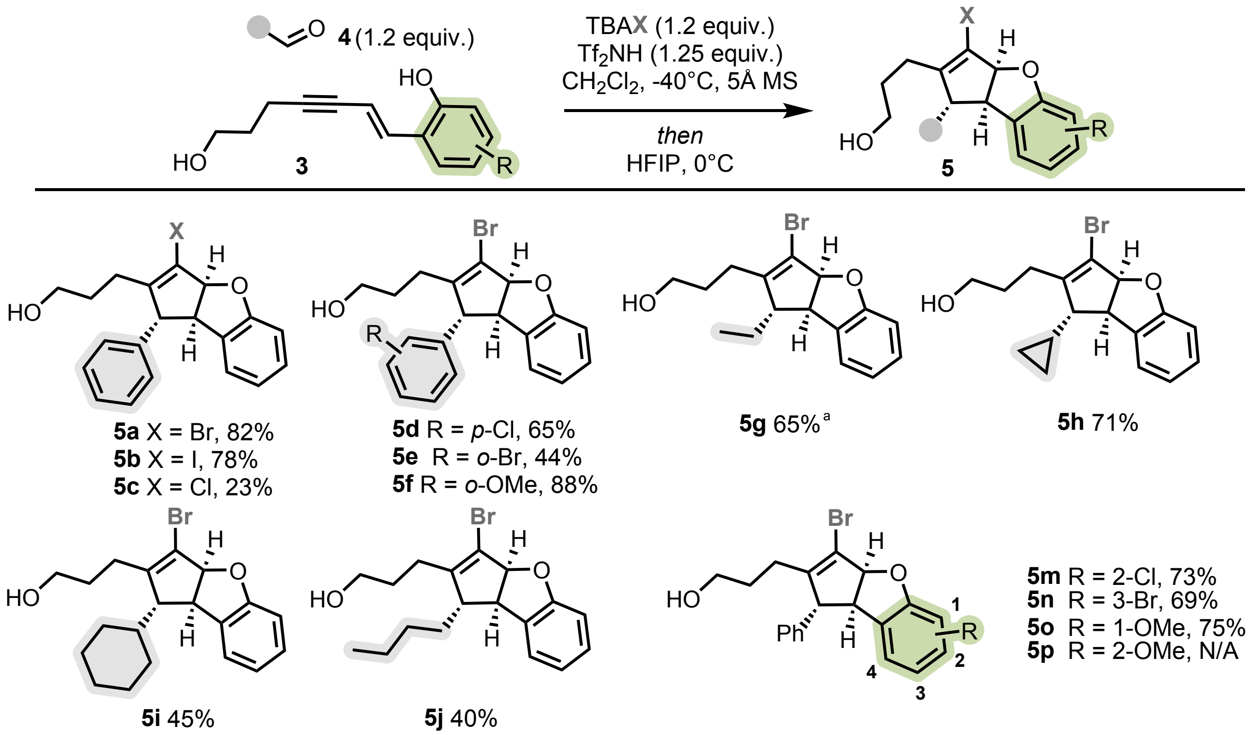

Halo-Prins/*Halo*-Nazarov one-pot; *General Procedure:* A round-bottom flask equipped with a stir bar, 5 Å powdered molecular sieves (300 mg/mmol enyne **3**), was closed with a septum and then flame-dried under vacuum. The flask was let to cool to room temperature and then refilled with argon. The septum was briefly removed and enyne **3** (0.2 mmol, 1.0 equiv.), aldehyde **4** (1.2 equiv.)[a], TBA**X** (**X**=desired halide, 1.2 equiv.) and anhydrous dichloromethane were added (2 mL). Care was taken to ensure TBA**X** dissolved, then the mixture was cooled to ~-40 °C using the dry ice/acetone bath. Once the mixture was cooled to -40 °C, bis(trifluoromethanesulfonyl)imide (1.25 equiv.) was added by weighing by difference and dipping spatula into the mix. (Bis(trifluoromethanesulfonyl)imide is a hygroscopic solid and therefore should be added under argon). Reaction mixture was left to stir at -40°C. Reaction progress was monitored using TLC (30% ethyl acetate/hexanes). Once full (or near full) consumption of enyne **3** into the *halo*-Prins intermediate was observed (typically around 10 minutes), the reaction flask was transferred to an ice bath to slowly warm up to 0°C. As the reaction was warmed up, HFIP (10 vol% of dichloromethane) was added dropwise to ensure consistent reaction temperature. The reaction was left to stir at 0°C until the *halo*-Prins intermediate was fully consumed into **5**. *Quench:* Sodium bicarbonate (solid) was added to the reaction mixture in excess. The mixture was diluted with ethyl acetate and the mixture was vacuum filtered through celite into a separatory funnel. The

celite was washed ethyl acetate. After filtering under vacuum, the organic layer was washed with sat. aq. sodium bicarbonate, then with brine. The organic layer was collected and dried by the addition of excess anhydrous magnesium sulfate (filtered over a fritted funnel under vacuum) and the solvents were removed by vacuum distillation using a rotary evaporator. The crude was purified by flash column chromatography to obtain the desired *halo*-Nazarov product **5** (SiO_2, 20% ethyl acetate/hexanes Rf ~ 0.2).

[a]2.0 equivalents of propionaldehyde was used. *Note: Keep products out of light, the vinyl halide moiety is light sensitive. Use aluminum foil to cover product when drying or rotavapping. These products should be stored at 0°C or they will begin to decompose (often starting to decompose after 2 hours at room temperature). NMRs may have ethyl acetate left over from purification due to product sensitivity to light and temperature.*

<u>Scope (Aldehydes)</u>

Compound 5a

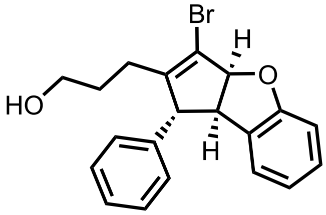

Compound **5a** was isolated as an off-white foam after column chromatography (gradient: 10% ethyl acetate/hexanes to 30% ethyl acetate/hexanes) in 82% yield (60 mg). Increasing the reaction scale from 0.2 mmol to 1.0 mmol gave **5a** in 82% yield (303 mg).

^{1}H NMR (400 MHz, $CDCl_3$) δ 7.39 (dd, *J* = 8.1, 6.6 Hz, 2H), 7.35 – 7.27 (m, 1H), 7.25 – 7.21 (m, 1H), 7.18 (ddd, *J* = 7.6, 5.1, 1.5 Hz, 3H), 6.95 – 6.86 (m, 2H), 5.87 (dt, *J* = 8.0, 2.0 Hz, 1H), 3.99 (d, *J* = 8.1 Hz, 1H), 3.94 (d, *J* = 2.1 Hz, 1H), 3.40 (q, *J* = 6.1 Hz, 2H), 2.35 (ddd, *J* = 13.9, 9.1, 7.2 Hz, 1H), 1.90 (dddd, *J* = 14.2, 9.0, 5.5, 1.9 Hz, 1H), 1.54 (tdd, *J* = 12.9, 8.1, 6.1 Hz, 1H), 1.41 (ddt, *J* = 13.5, 8.9, 6.7 Hz, 1H). **^{13}C NMR** ($CDCl_3$, 101 MHz): δ (ppm) 157.85, 148.18, 142.22, 129.88, 129.28,

129.14, 127.50, 127.44, 124.57, 121.19, 118.45, 110.77, 93.27, 62.12, 61.15, 53.16, 29.35, 25.04. **HRMS** (ESI-Orbitrap) m/z calc'd for $C_{20}H_{19}BrO_2$ [M+H]+ : 371.0647, found: 371.0644.

Compound 5b

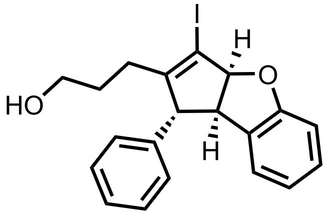

Compound **5b** has been made by our group before. See reference for full characterization data.[3]

Compound 5c

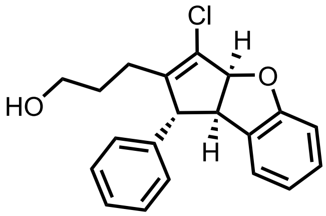

Compound **5c** was isolated as a yellow foam after column chromatography (gradient: 10% ethyl acetate/hexanes to 30% ethyl acetate/hexanes) in 23% yield (15 mg).

^{1}H NMR (400 MHz, $CDCl_3$) δ 7.39 (t, J = 7.4 Hz, 2H), 7.34 – 7.27 (m, 1H), 7.27 – 7.15 (m, 5H), 6.96 – 6.86 (m, 2H), 5.82 (dt, J = 8.3, 2.2 Hz, 1H), 4.01 – 3.93 (m, 2H), 3.41 (hept, J = 5.3, 4.5 Hz, 2H), 2.36 (dt, J = 14.1, 8.0 Hz, 1H), 1.89 (ddd, J = 14.3, 8.4, 6.1 Hz, 1H), 1.61 – 1.33 (m, 2H). **^{13}C NMR** ($CDCl_3$, 101 MHz): δ (ppm) 157.9, 144.7, 142.4, 129.9, 129.3, 129.1, 127.5, 127.5, 124.5, 121.2, 110.8, 91.9, 62.7, 60.6, 52.6, 29.3, 23.4. **HRMS** (ESI-Orbitrap) m/z calc'd for $C_{20}H_{19}ClO_2$ [M+H]+ : 327.1146, found: 327.1145.

Compound 5d

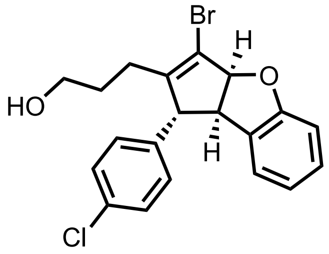

Compound **SI-9d** was isolated as a light yellow foam after column chromatography (gradient: 10% ethyl acetate/hexanes to 30% ethyl acetate/hexanes) in 65% yield (52 mg).

^{1}H NMR (400 MHz, $CDCl_3$) δ 7.38 – 7.31 (m, 2H), 7.23 – 7.14 (m, 2H), 7.14 – 7.06 (m, 2H), 6.94 – 6.84 (m, 2H), 5.83 (dt, *J* = 7.9, 2.1 Hz, 1H), 3.93 (s, 1H), 3.90 (d, *J* = 2.1 Hz, 1H), 3.43 – 3.38 (m, 2H), 2.34 (ddd, *J* = 13.8, 9.2, 7.1 Hz, 1H), 1.86 (dddd, *J* = 14.2, 9.2, 5.4, 1.9 Hz, 1H), 1.56 – 1.48 (m, 1H), 1.39 (ddd, *J* = 13.5, 6.8, 2.4 Hz, 1H).**^{13}C NMR** ($CDCl_3$, 101 MHz): δ (ppm) 157.8, 147.8, 140.7, 133.3, 129.5, 129.45, 129.28, 128.79, 124.50, 121.27, 118.89, 110.8, 93.1, 62.1, 60.5, 53.2, 29.3, 25.0. **HRMS** (ESI-Orbitrap) m/z calc'd for $C_{20}H_{18}BrClO_2$ [M+H]+ : 405.0251, found: 405.0246.

Compound 5e

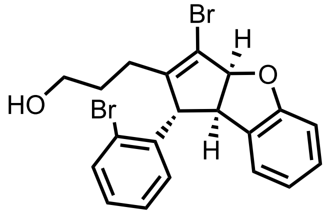

Compound **5e** was isolated as a light yellow foam after column chromatography (gradient: 10% ethyl acetate/hexanes to 30% ethyl acetate/hexanes) in 44% yield (39 mg).

^{1}H NMR (400 MHz, $CDCl_3$) δ 7.37 (t, *J* = 7.4 Hz, 2H), 7.29 (d, *J* = 19.0 Hz, 3H), 7.15 (d, *J* = 7.4 Hz, 2H), 5.86 (dd, *J* = 8.1, 2.0 Hz, 1H), 3.95 (d, *J* = 8.0 Hz, 1H), 3.89 (s, 1H), 3.42 (q, *J* = 6.2 Hz, 2H), 2.34 (dt, *J* = 15.3, 8.1 Hz, 1H), 1.89 (ddd, *J* = 14.3, 8.6, 5.7 Hz, 1H), 1.47 (ddq, *J* = 57.7, 14.5, 7.4 Hz, 3H).
^{13}C NMR($CDCl_3$, 101 MHz): δ (ppm) 157.11, 148.45, 141.74, 132.35, 131.98, 129.36, 127.67, 127.55, 127.37, 117.98, 112.84, 112.39, 93.99, 62.10, 61.06, 53.00,

29.80, 29.35, 25.04. **HRMS** (ESI-Orbitrap) m/z calc'd for $C_{20}H_{18}Br_2O_2$ [M+H]+ : 448.9746, found: 448.9749.

Compound 5f

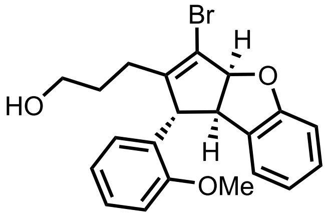

Compound **5f** was isolated as a bright yellow foam after column chromatography (30% ethyl acetate/hexanes) in 88% yield (70 mg).

^{1}H NMR (400 MHz, $CDCl_3$) δ 7.33 (d, J = 7.4 Hz, 1H), 7.30 – 7.25 (m, 1H), 7.16 (t, J = 7.7 Hz, 1H), 6.98 – 6.91 (m, 4H), 6.88 (t, J = 7.8 Hz, 1H), 5.74 (dt, J = 7.9, 2.1 Hz, 1H), 4.35 (s, 1H), 3.93 (s, 3H), 3.88 (d, J = 8.0 Hz, 1H), 3.38 (q, J = 6.0 Hz, 2H), 2.49 – 2.34 (m, 1H), 1.94 (ddt, J = 8.8, 5.3, 2.6 Hz, 1H), 1.53 (dtd, J = 12.7, 6.6, 6.2, 2.4 Hz, 1H), 1.43 (ddt, J = 13.5, 8.7, 6.6 Hz, 1H). **^{13}C NMR** ($CDCl_3$, 101 MHz): δ (ppm) 157.9, 157.2, 147.0, 130.4, 130.3, 128.9, 128.5, 124.9, 121.1, 121.0, 118.7, 110.9, 110.5, 93.2, 62.1, 55.6, 29.4, 25.1. **HRMS** (ESI-Orbitrap) m/z calc'd for $C_{21}H_{21}BrO_3$ [M+H]+ : 401.0752, found: 401.0724.

Compound 5g

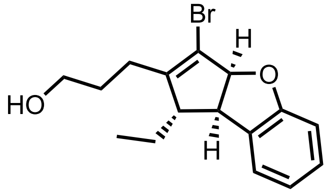

2.0 equivalents of propionaldehyde was used. Compound **5g** was isolated as a light yellow oil after column chromatography (gradient: 10% ethyl acetate/hexanes to 30% ethyl acetate/hexanes) in 65% yield (38 mg).

^{1}H NMR (400 MHz, $CDCl_3$) δ 7.11 (d, *J* = 7.4 Hz, 2H), 6.90 – 6.80 (m, 2H), 5.69 – 5.58 (m, 1H), 3.72 (d, *J* = 8.2 Hz, 1H), 3.48 (h, *J* = 4.5 Hz, 2H), 2.73 (d, *J* = 9.0 Hz, 1H), 2.40 (dt, *J* = 14.2, 8.3 Hz, 1H), 2.09 (ddd, *J* = 14.3, 9.3, 5.1 Hz, 1H), 1.84 (dtd, *J* = 14.7, 7.3, 3.1 Hz, 1H), 1.65 (dddd, *J* = 21.8, 15.6, 10.8, 5.3 Hz, 1H), 1.51 (dq, *J* = 9.4, 7.1 Hz, 1H), 1.38 (dq, *J* = 22.1, 7.4 Hz, 2H), 1.03 (t, *J* = 7.3 Hz, 3H). **^{13}C NMR**

(CDCl3, 101 MHz): δ (ppm) 158.0, 148.5, 130.5, 128.8, 124.5, 120.9, 116.9, 110.5, 93.1, 62.2, 55.8, 48.5, 29.0, 26.4, 24.6, 11.3. **HRMS** (ESI-Orbitrap) m/z calc'd for $C_{16}H_{19}BrO_2$ [M+H]+ : 323.0641, found: 323.0651.

Compound 5h

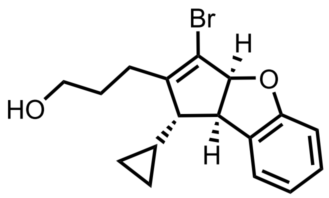

Compound **5h** was isolated as a yellow oil after column chromatography (20% ethyl acetate/hexanes) in 71% yield (47 mg).

^{1}H NMR (400 MHz, $CDCl_3$) δ 7.15 – 7.03 (m, 2H), 6.90 – 6.79 (m, 2H), 5.68 (d, J = 8.1 Hz, 1H), 3.91 (d, J = 8.1 Hz, 1H), 3.49 (t, J = 6.5 Hz, 2H), 2.39 (t, J = 7.9 Hz, 2H), 1.99 (d, J = 8.5 Hz, 1H), 1.70 (dp, J = 14.7, 7.5, 7.1 Hz, 1H), 1.57 (dq, J = 13.3, 8.6, 7.0 Hz, 1H), 0.73 (dq, J = 8.5, 4.8 Hz, 2H), 0.65 (dt, J = 12.1, 4.4 Hz, 1H), 0.44 (dd, J = 9.2, 5.0 Hz, 1H), 0.31 (dq, J = 9.7, 4.7 Hz, 1H). **^{13}C NMR** ($CDCl_3$,101 MHz): δ (ppm) 157.9, 149.4, 130.2, 128.9, 124.4, 120.9, 117.3, 110.6, 92.9, 62.2, 60.2, 50.6, 29.8, 25.1, 16.7, 6.1, 3.2. **HRMS** (ESI-Orbitrap) m/z calc'd for $C_{17}H_{19}BrO_2$ [M+H]+ : 335.0647, found: 335.0642.

Compound 5i

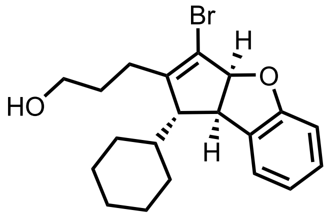

Compound **5i** was isolated as a yellow oil after column chromatography (gradient: 10% ethyl acetate/hexanes to 30% ethyl acetate/hexanes) in 45% yield (33 mg).

^{1}H NMR (400 MHz, $CDCl_3$) δ 7.14 – 7.04 (m, 2H), 6.88 – 6.81 (m, 2H), 5.54 (dt, *J* = 8.2, 2.0 Hz, 1H), 3.78 (d, *J* = 8.1 Hz, 1H), 3.47 (q, *J* = 5.8 Hz, 2H), 2.75 (s, 1H), 2.40 (ddd, *J* = 13.8, 9.1, 7.4 Hz, 1H), 2.10 – 2.04 (m, 1H), 1.90 – 1.83 (m, 1H), 1.71 (dddd, *J* = 29.4, 14.1, 9.6, 4.8 Hz, 6H), 1.55 – 1.31 (m, 6H), 0.84 (td, *J* = 12.5, 4.0 Hz, 1H). **^{13}C NMR** ($CDCl_3$, 101 MHz): δ (ppm) 157.9, 147.2, 130.9, 128.7, 124.4, 120.9, 116.9, 110.5, 93.3, 62.2, 60.3, 45.1, 38.9, 32.3, 29.4, 26.9, 26.5, 26.2, 24.7.

HRMS (ESI-Orbitrap) m/z calc'd for $C_{20}H_{25}BrO_2$ [M+H]+ : 377.1116, found: 377.1082.

Compound 5j

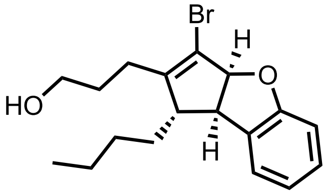

Compound **5j** was isolated as an off-white foam after column chromatography (gradient: 10% ethyl acetate/hexanes to 30% ethyl acetate/hexanes) in 40% yield (29 mg).

^{1}H NMR (400 MHz, $CDCl_3$) δ 7.17 – 7.07 (m, 2H), 6.91 – 6.80 (m, 2H), 5.62 (dt, J = 8.2, 2.2 Hz, 1H), 3.73 (d, J = 8.1 Hz, 1H), 3.48 (td, J = 6.4, 3.9 Hz, 2H), 2.75 (dt, J = 9.0, 2.1 Hz, 1H), 2.41 (ddd, J = 13.9, 9.2, 7.2 Hz, 1H), 2.10 (dddd, J = 14.4, 9.3, 5.3, 2.0 Hz, 1H), 1.81 – 1.60 (m, 3H), 1.58 – 1.21 (m, 9H), 0.96 (t, J = 6.5 Hz, 3H).**^{13}C NMR** ($CDCl_3$, 101 MHz): δ (ppm) 158.0, 148.8, 130.5, 128.8, 124.5, 120.9, 116.7, 110.5, 93.1, 62.2, 54.5, 49.0, 33.5, 29.5, 24.6, 22.9, S15 14.2. **HRMS** (ESI-Orbitrap) m/z calc'd for $C_{18}H_{23}BrO_2$ [M+H]+ : 351.0954, found: 351.0956.

Scope (Enyne Substituents)

Compound 5m

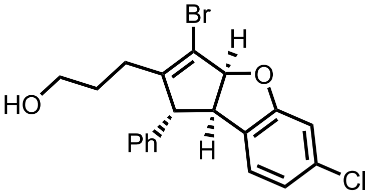

Compound **5m** was isolated as a yellow oil after column chromatography (gradient: 10% ethyl acetate/hexanes to 30% ethyl acetate/hexanes) in 73% yield (38 mg).

^{1}H NMR (400 MHz, $CDCl_3$) δ 7.41 – 7.34 (m, 2H), 7.29 (td, *J* = 7.4, 1.4 Hz, 1H), 7.19 – 7.08 (m, 4H), 6.79 (dd, *J* = 8.6, 1.4 Hz, 1H), 5.87 (dq, *J* = 8.1, 1.8 Hz, 1H),

3.94 (d, J = 8.1 Hz, 1H), 3.89 (d, J = 2.4 Hz, 1H), 3.41 (q, J = 5.4 Hz, 2H), 2.38 – 2.29 (m, 1H), 1.88 (ddd, J = 14.3, 8.3, 5.2 Hz, 1H), 1.59 – 1.49 (m, 1H), 1.39 (dtd, J = 9.0, 7.6, 6.0 Hz, 1H). **^{13}C NMR** ($CDCl_3$, 101 MHz): δ (ppm) 156.6, 148.4, 141.8, 131.8, 129.4, 129.1, 127.7, 127.4, 125.8, 124.7, 118.0, 111.8, 94.0, 62.1, 61.0, 53.1, 29.3, 25.0. **HRMS** (ESI-Orbitrap) m/z calc'd for $C_{20}H_{18}BrClO_2$ [M+H]+ : 405.0789, found: 405.0856.

Compound 5n

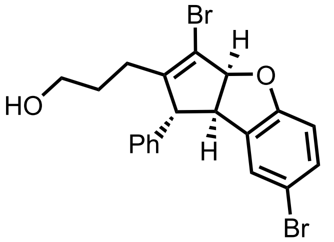

Compound **5n** was isolated as a yellow foam after column chromatography (gradient: 10% ethyl acetate/hexanes to 30% ethyl acetate/hexanes) in 69% yield (62 mg).

^{1}H NMR (400 MHz, $CDCl_3$) δ 7.37 (t, J = 7.4 Hz, 2H), 7.30 – 7.20 (m, 2H), 7.14 (d, J = 7.4 Hz, 2H), 6.75 (dd, J = 8.4, 1.3 Hz, 1H), 5.86 (dd, J = 8.1, 2.0 Hz, 1H), 3.95 (d, J = 8.0 Hz, 1H), 3.88 (d, J = 2.5 Hz, 1H), 3.41 (q, J = 5.9 Hz, 2H), 2.33 (dt, J = 15.2, 8.1 Hz, 1H), 1.88 (ddd, J = 14.3, 7.5, 5.2 Hz, 1H), 1.60 – 1.49 (m, 1H), 1.45 – 1.33 (m, 2H). **^{13}C NMR** ($CDCl_3$, 101 MHz): δ (ppm) 157.1, 148.5, 141.7, 132.5, 131.9, 129.4, 127.7, 127.6, 127.4, 127.4, 117.9, 112.9, 112.4, 93.9, 62.1, 61.1, 53.0, 29.3, 25.0. **HRMS** (ESI-Orbitrap) m/z calc'd for $C_{20}H_{18}Br_2O_2$ [M+H]+ : 448.9746, found: 448.9766

Compound 5o

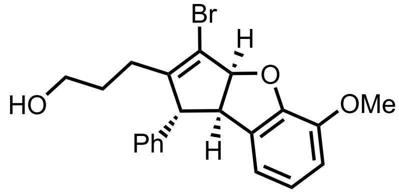

Compound **5o** was isolated as a yellow oil after column chromatography (gradient: 10% ethyl acetate/hexanes to 30% ethyl acetate/hexanes) in 75% yield (60 mg).

^{1}H NMR (400 MHz, CDCl3) δ 7.37 (t, J = 7.4 Hz, 2H), 7.32 – 7.26 (m, 1H), 7.16 (d, J = 7.5 Hz, 2H), 6.90 – 6.82 (m, 2H), 6.79 – 6.75 (m, 1H), 5.90 (dt, J = 8.1, 2.1 Hz, 1H), 3.99 (d, J = 8.1 Hz, 1H), 3.92 (s, 1H), 3.88 (d, J = 1.1 Hz, 3H), 3.47 – 3.36 (m, 2H), 2.34 (dt, J = 15.3, 8.1 Hz, 1H), 1.96 – 1.83 (m, 1H), 1.50 (d, J = 6.3 Hz, S16 1H), 1.40 (dt, J = 14.3, 7.3 Hz, 1H).**^{13}C NMR** ($CDCl_3$, 101 MHz): δ (ppm) 148.1, 146.2, 145.4, 142.2, 131.0, 127.5, 127.4, 121.8, 118.4, 116.5, 112.1, 93.8, 62.2, 60.9, 56.1, 53.7, 29.4, 25.1. **HRMS** (ESI-Orbitrap) m/z calc'd for $C_{21}H_{21}BrO_3$ [M+H]+ : 401.0747, found: 401.0749.

<u>Optimization Table: Solvent Screening</u>

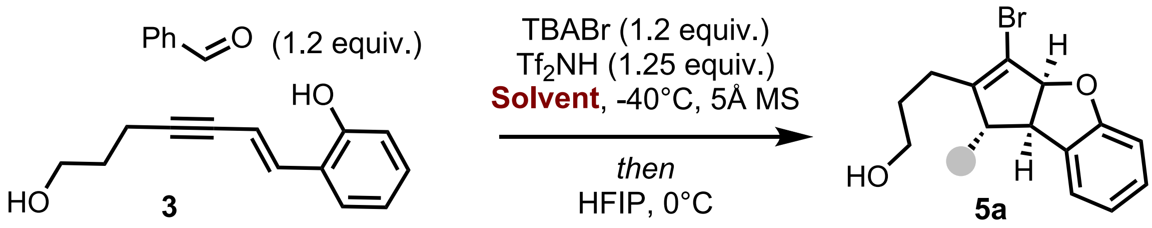

#	Solvent	Temperature	Total Time	Yield (%)
**1**	DCM	-40°C	20 minutes	82
**2**	DCM/TFE (10:1)	-40°C	20 minutes	36
**3**	DCM	-20°C	30 minutes	28
**4**	DCE	-30°C	25 minutes	62
**5**	Toluene[a]	-40°C	3 hours	57
**6**	Toluene	-40°C	24 hours	27
**7**	Toluene/HFIP (10:1)	0°C to 25 °C	24 hours	23
**8**	Trifluorotoluene /DCM (20:1)	-25°C	1 hour, 10 minutes	51
**9**	1,4-Dichlorobenzene	-20°C	10 minutes	12
**10**	Chlorobenzene	-40°C	40 minutes	44
**11**	Dimethoxyethane	-40°C	24 hours	[b]

Table 2: Solvent Screening of *Halo*-Prins/*Halo*-Nazarov Reaction cascade.

[a] 0.5 mL of DCM added for solubility of **3**. [b] No product observed.

General procedure for *halo*-Prins/*halo*-Nazarov on page S11-S12 used for solvent screening, only varying the solvent.

General Reaction Scheme for *Halo*-Prins/*Halo*-Nazarov Cascade of Benzyl Enynes

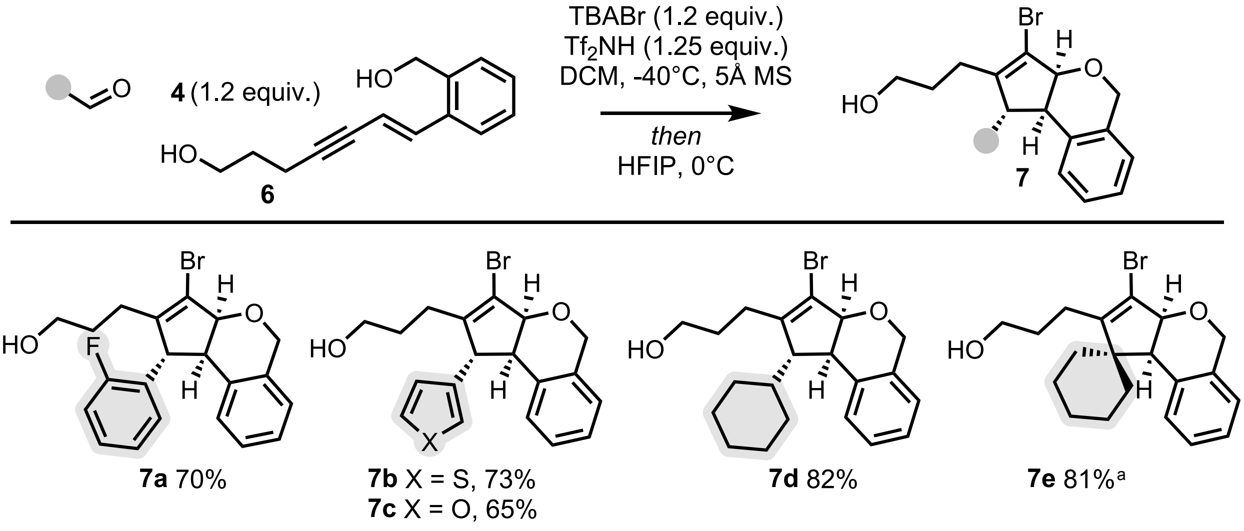

Halo-Prins/Halo-Nazarov one-pot; General Procedure: A 5 mL round-bottom flask equipped with a stir bar, 5 Å powdered molecular sieves (300 mg/mmol enyne **3**), was closed with a septum and then flame-dried under vacuum. The flask was let to cool to room temperature and then refilled with argon. The septum was briefly removed and enyne **6** (0.2 mmol, 1.0 equiv.), carbonyl compound **4** (1.2 equiv.), TBABr (1.2 equiv.) and anhydrous, unstabilized dichloromethane was added (2 mL). Care was taken to ensure TBABr dissolved, then the mixture was cooled to -40 °C using the dry ice/acetone bath. Once the mixture was cooled to -40 °C, bis(trifluoromethanesulfonyl)imide (1.25 equiv.) was added by weighing by difference and dipping spatula into the reaction mixture. Reaction mixture was left to stir at -40°C. Reaction progress was monitored using TLC (30% ethyl acetate/hexanes). Once full (or near full) consumption of enyne **6** into the *halo*-Prins intermediate was observed (typically around 5 minutes), the reaction flask was transferred to an ice bath to slowly warm up to 0°C. As the reaction was warming up, HFIP (10 vol% of DCM) was added dropwise to ensure consistent reaction mixture temperature. The reaction was left to stir at 0°C until the *halo*-Prins intermediate was fully consumed into **7a-e**. *Quench:* Sodium bicarbonate (solid/powder) was added to the reaction mixture in excess. The mixture was diluted with ethyl acetate and the mixture was vacuum filtered through celite into a separatory funnel. The celite was washed with ethyl acetate. After filtering under vacuum, the organic layer was washed with sat. aq. sodium bicarbonate, then with brine. The organic layer was collected and dried by the addition of excess anhydrous

magnesium sulfate (filtered over a fritted funnel under vacuum) and the solvents were removed by vacuum distillation using a rotary evaporator. The crude was purified by flash column chromatography to obtain the desired *halo*-Nazarov product **7a-d** (SiO_2, 30% ethyl acetate/hexanes Rf ~ 0.18).

[a] 2.0 equivalents of cyclohexanone used.

Compound 7a

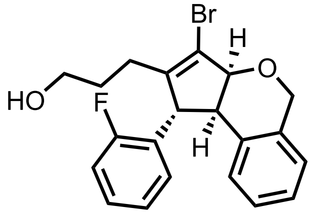

Compound **7a** was isolated as a yellow foam after column chromatography (gradient: 10% ethyl acetate/hexanes to 30% ethyl acetate/hexanes) in 70% yield (56 mg).

^{1}H NMR (400 MHz, $CDCl_3$) δ 7.30 (ddt, *J* = 8.5, 5.3, 2.6 Hz, 1H), 7.21 – 7.02 (m, 6H), 6.80 (d, *J* = 7.5 Hz, 1H), 4.91 (dd, *J* = 6.4, 1.6 Hz, 1H), 4.83 – 4.57 (m, 2H), 4.13 (d, *J* = 6.3 Hz, 1H), 3.52 – 3.46 (m, 2H), 2.45 (dt, *J* = 13.7, 8.1 Hz, 1H), 1.85 – 1.75 (m, 1H), 1.63 – 1.50 (m, 1H), 1.46 (dt, *J* = 13.7, 6.9 Hz, 2H). **^{13}C NMR** ($CDCl_3$, 101 MHz): δ (ppm) 162.4, 159.9, 148.7, 134.9, 134.6, 129.2, 129.1, 128.1, 127.4, 126.5, 124.9, 124.9, 124.7, 118.8, 116.3, 116.1, 83.9, 66.0, 62.1, 29.3, 25.0. **HRMS** (ESI-Orbitrap) m/z calc'd for $C_{21}H_{20}BrFO_2$ [M+H]+ : 403.0701, found: 403.0685.

Compound 7b

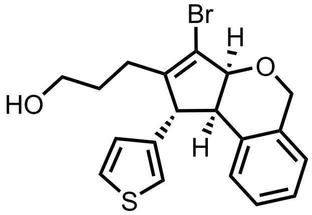

Compound **7b** was isolated as a yellow oil after column chromatography (30% ethyl acetate/hexanes) in 73% yield (56 mg).

^{1}H NMR (400 MHz, CDCl3) δ 7.30 (d, J = 5.1 Hz, 1H), 7.17 (t, J = 7.4 Hz, 1H), 7.10 (t, J = 7.4 Hz, 1H), 7.05 (d, J = 7.5 Hz, 1H), 7.01 (t, J = 4.4 Hz, 1H), 6.90 (dd,

J = 11.6, 5.5 Hz, 2H), 4.86 – 4.81 (m, 1H), 4.79 – 4.63 (m, 2H), 4.12 (d, J = 6.8 Hz, S20 1H), 3.52 (p, J = 4.4, 3.7 Hz, 2H), 3.47 (t, J = 6.6 Hz, 1H), 2.41 (dt, J = 13.5, 8.1 Hz, 1H), 2.01 – 1.99 (m, 1H), 1.94 (td, J = 8.1, 4.6 Hz, 1H), 1.59 (h, J = 7.1 Hz, 2H), 1.49 (dt, J = 13.9, 7.0 Hz, 1H).^{13}**C NMR** ($CDCl_3$, 101 MHz): δ (ppm) 149.6, 145.3,134.5, 128.2, 127.3, 127.3, 126.6, 126.2, 125.2, 124.6, 118.8, 83.7, 66.5, 62.2, 54.9, 48.4, 29.6, 25.0. **HRMS** (ESI-Orbitrap) m/z calc'd for $C_{19}H_{19}BrO_2S$ [M+H]+ :391.0645, found: 391.0695.

Compound 7c

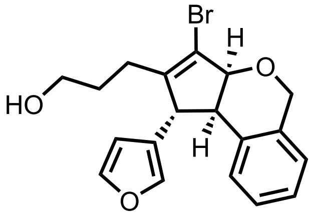

Compound **7c** was isolated as a yellow oil after column chromatography (30% ethyl acetate/hexanes) in 55% yield (41 mg).

^{1}**H NMR** (400 MHz, $CDCl_3$) δ 7.45 (s, 1H), 7.20 – 7.09 (m, 2H), 7.05 (d, *J* = 7.2 Hz, 1H), 6.83 (d, *J* = 7.4 Hz, 1H), 6.39 (dt, *J* = 3.1, 1.5 Hz, 1H), 6.22 (d, *J* = 3.1 Hz, 1H), 4.81 (dd, *J* = 6.1, 1.5 Hz, 1H), 4.78 – 4.59 (m, 2H), 3.90 (d, *J* = 7.1 Hz, 1H), 3.61 (t, *J* = 6.7 Hz, 1H), 3.53 (tt, *J* = 6.4, 3.7 Hz, 2H), 1.95 (ddd, *J* = 13.9, 8.4, 6.2 Hz, 1H), 1.54 (dt, *J* = 14.1, 7.3 Hz, 2H), 1.44 (dq, *J* = 14.1, 7.5, 7.1 Hz, 1H).^{13}**C NMR** ($CDCl_3$, 101 MHz): δ (ppm) 153.5, 147.9, 142.6, 134.6, 134.5, 128.3, 127.4, 126.5, 124.6, 118.8, 110.7, 108.4, 83.8, 66.7, 62.2, 52.9, 43.4, 29.4, 25.2. **HRMS** (ESI-Orbitrap) m/z calc'd for $C_{19}H_{19}BrO_2S$ [M+H]+ : 375.0596, found: 375.0557.

Compound 7d

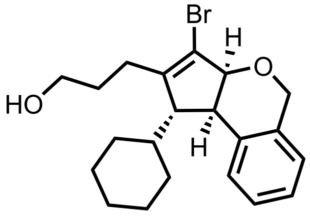

Compound **7d** was isolated as a yellow oil after column chromatography (gradient: 10% ethyl acetate/hexanes to 30% ethyl acetate/hexanes) in 82% yield (66 mg).

^{1}H NMR (400 MHz, $CDCl_3$) δ 7.45 (d, *J* = 7.2 Hz, 1H), 7.41 – 7.37 (m, 1H), 7.30 (ddt, *J* = 8.8, 3.8, 2.1 Hz, 3H), 6.93 (d, *J* = 14.7 Hz, 1H), 4.79 (d, *J* = 4.0 Hz, 2H), 4.49 (d, *J* = 9.8 Hz, 1H), 3.77 (td, *J* = 10.9, 4.6 Hz, 1H), 3.63 (dt, *J* = 11.8, 3.8 Hz, 1H), 3.16 (dt, *J* = 14.3, 3.9 Hz, 1H), 2.33 (td, *J* = 13.2, 6.8 Hz, 1H), 2.08 – 1.99 (m, 3H), 1.85 – 1.60 (m, 7H), 1.47 – 1.39 (m, 1H), 1.16 (ddt, *J* = 19.8, 12.1, 6.5 Hz, 4H). **^{13}C NMR** ($CDCl_3$, 101 MHz): δ (ppm) 140.6, 138.3, 135.8, 131.9, 128.6, 128.4, 128.2, 126.4, 125.5, 120.5, 79.8, 63.5, 60.9, 37.51, 29.9, 29.5, 29.4, 27.5, 26.4, 26.3, 26.0. **HRMS** (ESI-Orbitrap) m/z calc'd for $C_{21}H_{27}BrO_2$ [M+H]+ : 391.1273, found: 391.0347.

Compound 7e

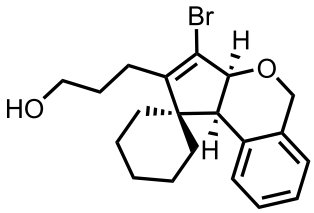

Compound **7e** was isolated as a yellow foam after column chromatography (gradient: 10% ethyl acetate/hexanes to 30% ethyl acetate/hexanes) in 81% yield (66 mg).

^{1}H NMR (400 MHz, $CDCl_3$) δ 7.36 (d, *J* = 6.8 Hz, 1H), 7.23 – 7.17 (m, 2H), 7.07 (d, *J* = 6.2 Hz, 1H), 4.64 (d, *J* = 6.6 Hz, 1H), 4.62 – 4.45 (m, 2H), 3.71 (t, *J* = 6.4 Hz, 2H), 3.17 (d, *J* = 6.6 Hz, 1H), 2.46 – 2.35 (m, 2H), 1.89 – 1.69 (m, 6H), 1.44 (ddd, *J* = 14.5, 9.2, 4.2 Hz, 1H), 1.23 (dq, *J* = 12.9, 6.5, 5.9 Hz, 1H), 1.02 (tq, *J* = 12.7, 4.4 Hz, 2H). **^{13}C NMR** ($CDCl_3$, 101 MHz): δ (ppm) 156.2, 137.5, 133.6, 131.1, 126.6, 126.5, 125.1, 117.9, 83.1, 65.2, 63.0, 53.9, 50.4, 37.5, 32.9, 31.7, 25.8, 25.1, 22.8, 21.7. **HRMS** (ESI-Orbitrap) m/z calc'd for $C_{20}H_{25}BrO_2$ [M+H]+ : 377.1111, found: 377.1123.

Product Derivatization

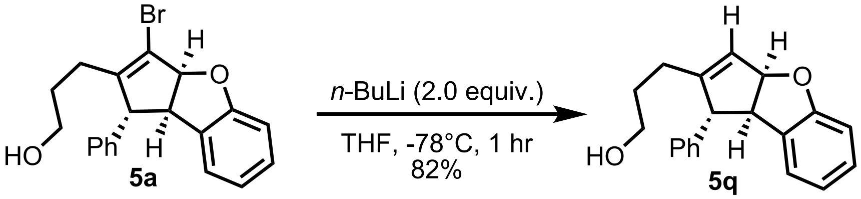

Dehalogenation: To a 50 mL round-bottom flask, a stir bar was added. The flask was flame-dried under vacuum, left to cool to room temperature under vacuum, then left under argon. The flask was charged with **5a** (0.28 mmol, 1.0 equiv.) and THF (5 mL). The reaction mixture was cooled to -78°C using an acetone/dry ice bath. Once cooled, *n*-BuLi (2.5 M in hexanes, 4.0 equiv.) was added dropwise over one minute. The reaction mixture was left to stir at -78°C for one hour. Once **5a** was consumed, H_2O (5.0 equiv.) was added at -78°C, after which the mixture was left to warm to rt. Once the mixture was warmed to room temperature, it was diluted with diethyl ether and poured into a separatory funnel. The organic layer was collected and dried by the addition of excess anhydrous magnesium sulfate (filtered over a fritted funnel under vacuum) and the solvents were removed by vacuum distillation using a rotary evaporator. The crude was purified by flash column chromatography to obtain the desired coupling product **5q** (Rf ~ 0.2 in 30% ethyl acetate/hexanes, stains a purple-blue in *p*-anisaldehyde; SiO_2, 20% ethyl acetate/hexanes) as an off-white foam in 82% yield. (67 mg)

^{1}H NMR (400 MHz, $CDCl_3$) δ 7.36 (t, *J* = 7.5 Hz, 2H), 7.30 – 7.20 (m, 3H), 7.14 (dd, *J* = 16.7, 7.7 Hz, 3H), 6.87 (t, *J* = 7.4 Hz, 1H), 6.79 (d, *J* = 8.0 Hz, 1H), 5.97 (dt, *J* = 8.1, 1.9 Hz, 1H), 5.73 (t, *J* = 1.8 Hz, 1H), 3.97 (d, *J* = 8.0 Hz, 1H), 3.89 (s, 1H), 3.47 (td, *J* = 6.5, 2.4 Hz, 2H), 1.93 (q, *J* = 8.1 Hz, 2H), 1.73 – 1.56 (m, 2H). **^{13}C NMR**($CDCl_3$, 101 MHz): δ (ppm) 158.3, 152.8, 143.5, 130.8, 129.1, 128.8, 127.5, 127.0, 124.9, 124.8, 120.6, 110.3, 92.0, 63.2, 62.4, 54.5, 30.1, 25.8. **HRMS** (ESI-Orbitrap) m/z calc'd for $C_{20}H_{20}O_2$ [M+H]+ : 293.1536, found: 293.1539.

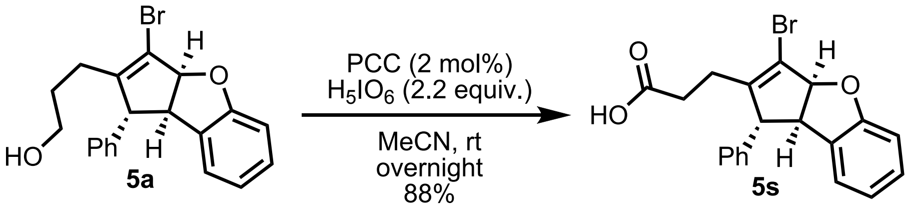

Oxidation: To a 25 mL round-bottom flask, a stir bar was added. The flask was flame-dried under vacuum, left to cool to room temperature under vacuum, then left under argon. The flask was charged with H_5IO_6 (2.2 equiv.) and MeCN (2 mL). The mixture was left to stir at room temperature for 15 minutes, then was cooled to 0°C using an ice/water bath. **5a** (0.2 mmol, 1.0 equiv.) was added, followed by PCC (2 mol%). After 15 minutes, the mixture was left to stir at room temperature for 3 hours. *Quench*: The reaction mixture was diluted with ethyl acetate and transferred to a separatory funnel. The organic layer was washed with excess water, which was then back-extracted with ethyl acetate 3x. All the organic layers were collected and washed with brine. The organic layer was collected and dried by the addition of excess anhydrous magnesium sulfate (filtered over a fritted funnel under vacuum) and the solvents were removed by vacuum distillation using a rotary evaporator to obtain crude **5s** as a clear oil in 88% yield (67 mg). (Rf ~ 0.2, stains a yellow-brown with *p*-anisaldehyde).

Caution: Thorough aqueous workup is essential to remove residual oxidant. Incomplete removal may result in explosive decomposition when placing the crude material under vacuum.

^{1}H NMR (400 MHz, $CDCl_3$) δ 7.79 (s, 1H), 7.36 (t, *J* = 7.4 Hz, 3H), 7.28 (t, *J* = 7.3 Hz, 3H), 7.20 (d, *J* = 7.4 Hz, 2H), 7.15 (d, *J* = 7.6 Hz, 5H), 6.91 – 6.83 (m, 3H), 5.83 (dd, *J* = 8.3, 2.3 Hz, 2H), 3.96 (d, *J* = 8.2 Hz, 2H), 3.89 (d, *J* = 2.2 Hz, 2H), 2.49 (ddd, *J* = 12.4, 10.0, 4.9 Hz, 2H), 2.27 (ddd, *J* = 15.2, 10.1, 4.3 Hz, 2H), 2.21 – 2.03 (m, 4H).**^{13}C NMR** ($CDCl_3$, 101 MHz): δ (ppm) 177.69, 157.82, 146.35, 141.82, 129.67, 129.40, 129.17, 127.67, 127.38, 124.57, 121.24, 119.46, 110.75, 93.83, 93.11, 61.41, 53.23, 30.73, 23.91. **HRMS** (ESI-Orbitrap) m/z calc'd for $C_{20}H_{17}BrO_3$ [M+H]+ : 385.0439, found: 385.0616.

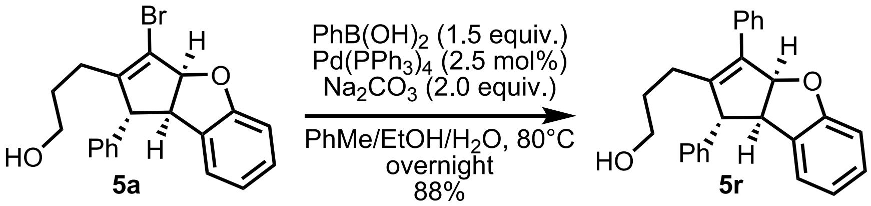

Suzuki Coupling: To a 5 mL pressure vial a stir bar was added. The flask was charged with **5a** (0.2 mmol, 1.0 equiv.), $PhB(OH)_2$ (1.5 equiv.), Na_2CO_3 (2.0 equiv.), PhMe (0.6 mL), EtOH (0.2 mL), H_2O (0.1 mL). A septum pierced with a long needle and bleed needle was placed on the pressure vial, with an Argon balloon connected to the long needle to degas the reaction mixture. After 30 minutes of continuous degassing, the degassing needle was removed, and $Pd(PPh_3)_4$ (2.5 mol%) was added. The pressure vial was closed with a white screw-top cap, and placed in an oil bath preheated to 80 °C. The reaction was left to stir at 80 °C overnight. *Quench*: Reaction mixture was left to cool to room temperature. The mixture was diluted with ethyl acetate and the mixture was vacuum filtered through celite into a separatory funnel. The celite was washed with ethyl acetate. After filtering under vacuum, the organic layer was washed with sat. aq. sodium bicarbonate, then with brine. The organic layer was collected and dried by the addition of excess anhydrous magnesium sulfate (filtered over a fritted funnel under vacuum) and the solvents were removed by vacuum distillation using a rotary evaporator. The crude was purified by flash column chromatography to obtain the desired coupling product **5r** as a bright yellow oil in 85% yield (64 mg). (Rf ~0.4 in 30% ethyl acetate/hexanes; SiO_2, 10% ethyl acetate/hexanes).

^{1}H NMR (400 MHz, $CDCl_3$) δ 7.41 (dt, *J* = 21.3, 7.4 Hz, 6H), 7.35 – 7.22 (m, 6H), 7.14 (t, *J* = 7.7 Hz, 1H), 6.89 (t, *J* = 7.4 Hz, 1H), 6.81 (d, *J* = 8.0 Hz, 1H), 6.17 (dt, *J* = 8.1, 1.8 Hz, 1H), 4.11 (s, 1H), 3.98 (d, *J* = 8.0 Hz, 1H), 3.32 (q, *J* = 6.7 Hz, 2H), 2.31 (ddd, *J* = 14.2, 9.5, 7.2 Hz, 1H), 1.83 (ddd, *J* = 14.5, 9.4, 5.2 Hz, 1H), 1.61 – 1.46 (m, 1H), 1.46 – 1.33 (m, 2H).**^{13}C NMR**($CDCl_3$, 101 MHz): δ (ppm) 158.41, 146.26, 143.71, 137.25, 129.19, 128.78, 128.56, 127.62, 127.56, 127.11, 124.68, 120.69, 110.41, 94.80, 62.54, 62.35, 53.04, 30.27, 23.79. **HRMS** (ESI-Orbitrap) m/z calc'd for $C_{26}H_{24}O_2$ [M+H]+ : 369.0439, found: 369.0616.

References

Eur. J. Org. Chem., 2013: 781-788.[1]

https://www.chem.rochester.edu/notvoodoo/pages/workup/aluminum_hydride_reduction.php[2]

Milosavlijevic, A. (2024) Halo-Prins/halo-Nazarov fragment coupling cationic cascades I. Nitrogen-interruption : synthesis of indolines II. Arene-interruption : total synthesis of Tubingensin A [Doctoral dissertation, University of Rochester]. [Link: http://hdl.handle.net/1802/38298][3]

NMR Spectra

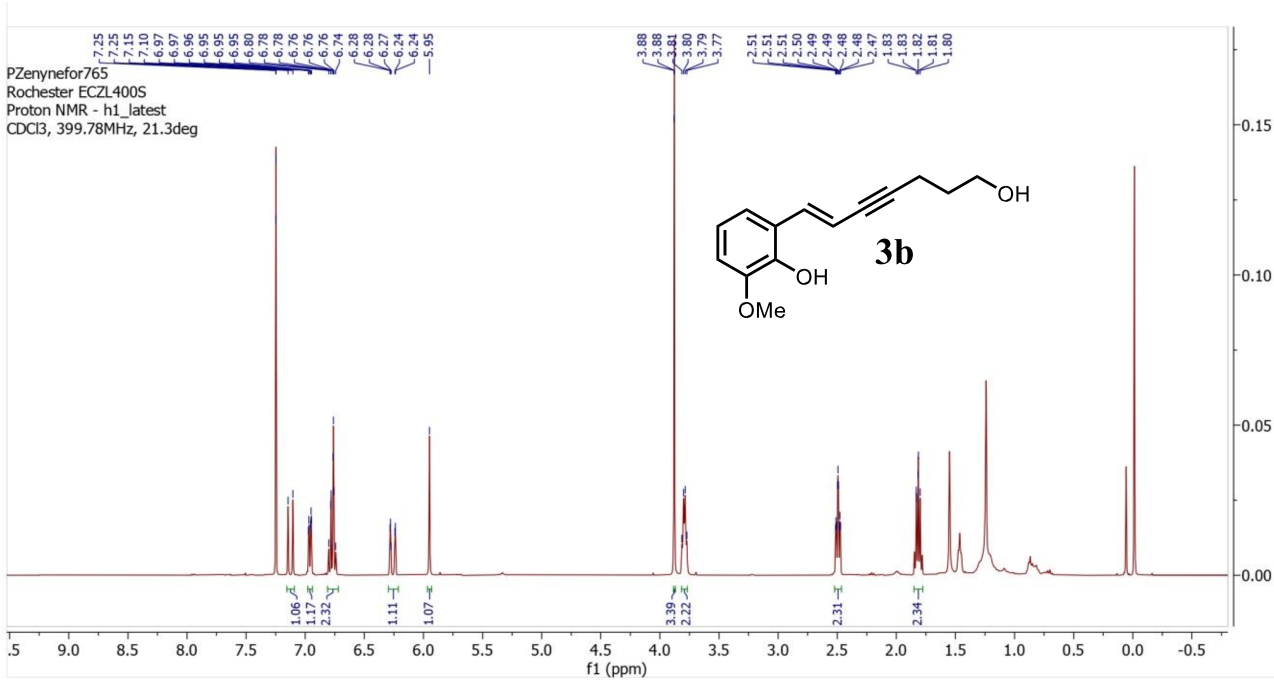
PZenynefor765
Rochester ECZL400S
Proton NMR - h1_latest
CDCl3, 399.78MHz, 21.3deg
OH
OH
OMe
3b
f1 (ppm)

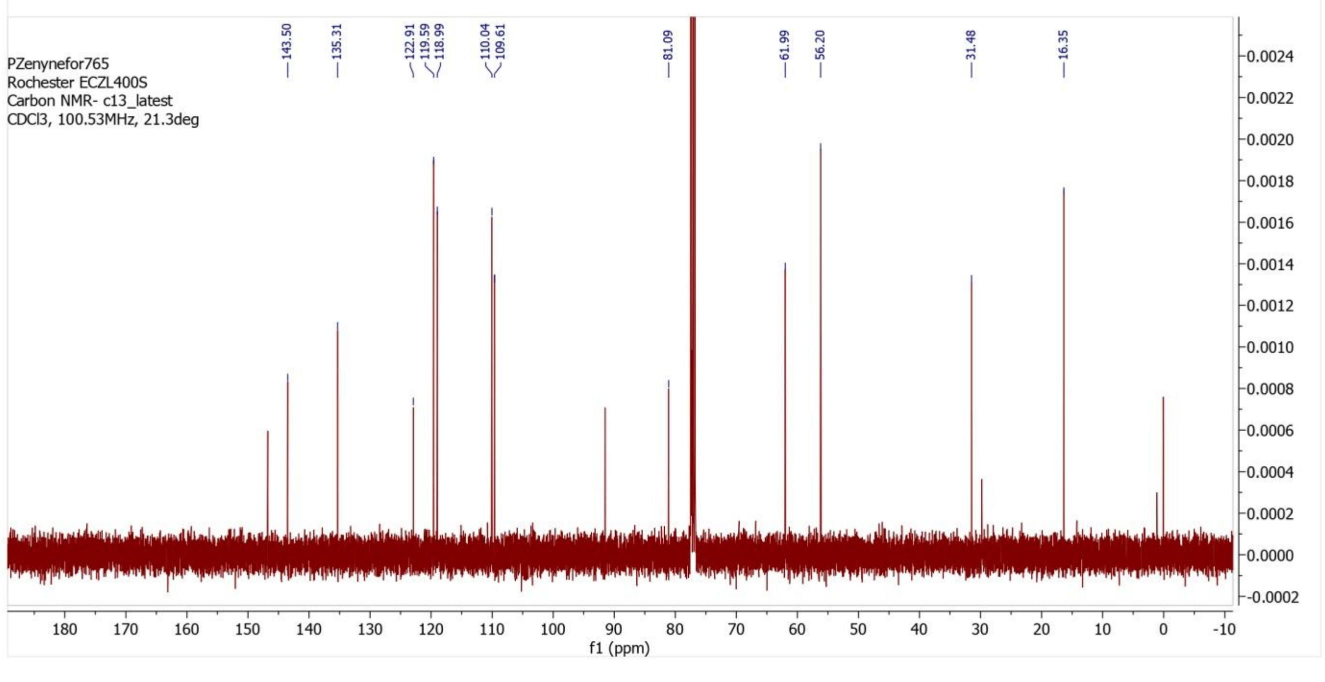
PZenynefor765
Rochester ECZL400S
Carbon NMR- c13_latest
CDCl3, 100.53MHz, 21.3deg
143.50
135.31
122.91
119.59
118.99
110.04
109.61
81.09
61.99
56.20
31.48
16.35
f1 (ppm)

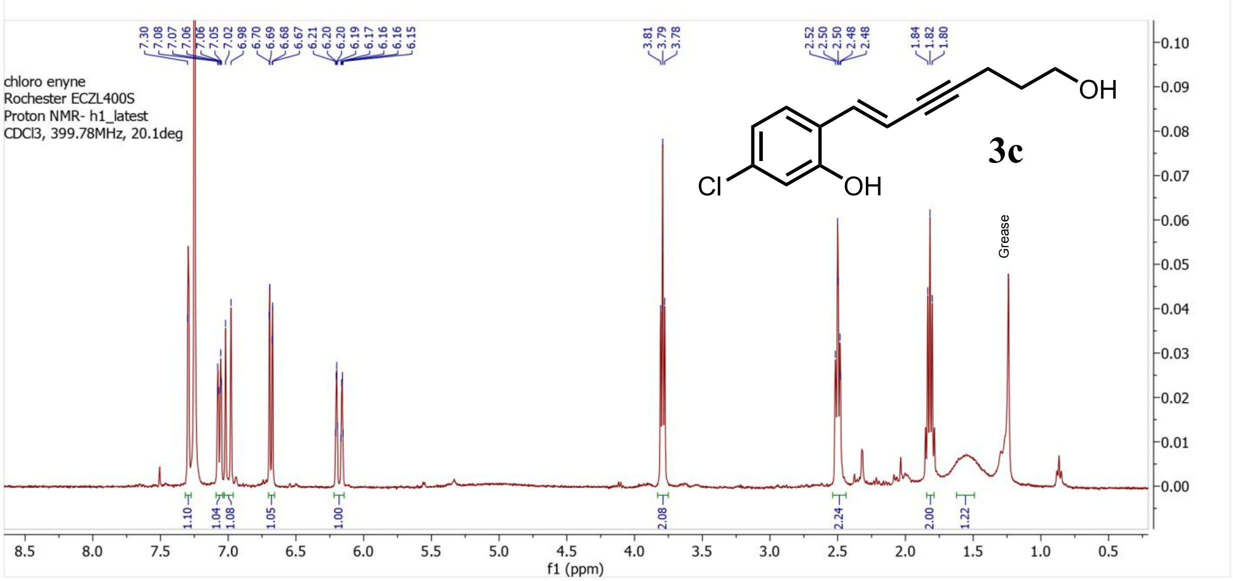
chloro enyne
Rochester ECZL400S
Proton NMR- h1_latest
CDCl3, 399.78MHz, 20.1deg
OH
Cl
OH
3c
Grease
f1 (ppm)

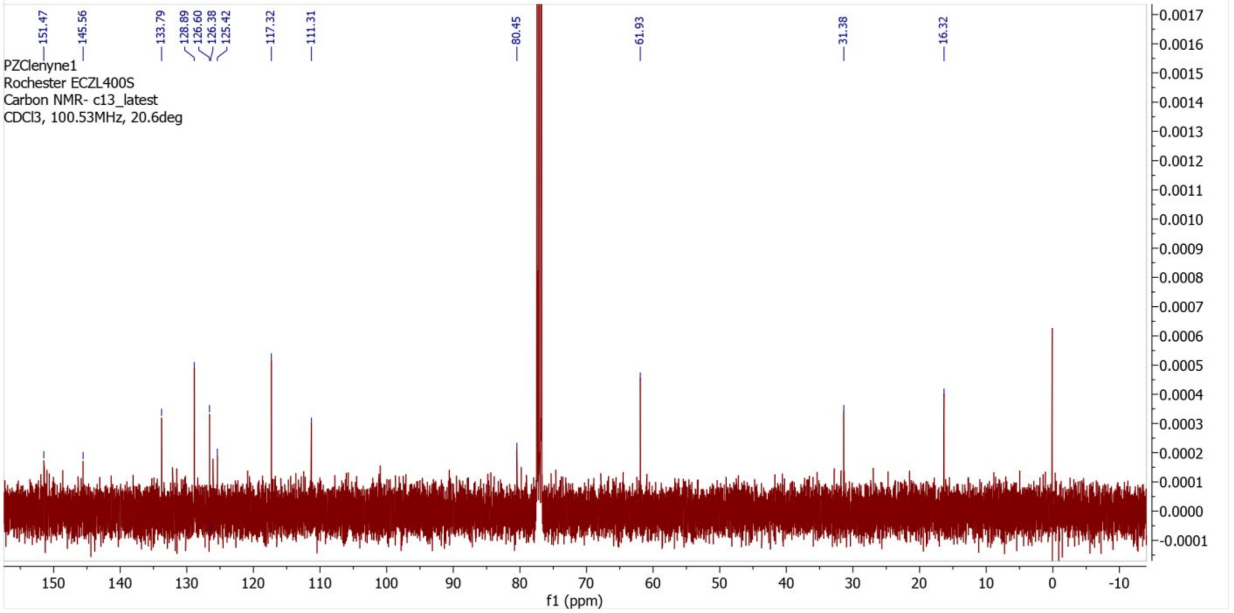
PZClenyne1
Rochester ECZL400S
Carbon NMR- c13_latest
CDCl3, 100.53MHz, 20.6deg
151.47
145.56
133.79
128.89
126.60
126.38
125.42
117.32
111.31
80.45
61.93
31.38
16.32
f1 (ppm)

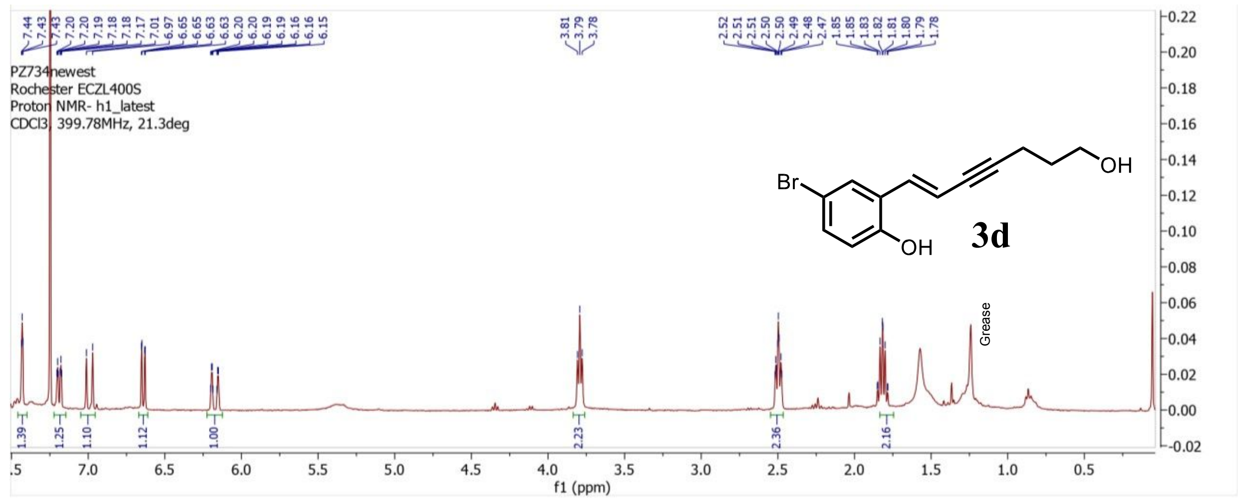
PZ734newest
Rochester ECZL400S
Proton NMR- h1_latest
CDCl3, 399.78MHz, 21.3deg
Br
OH
OH
3d
Grease
f1 (ppm)

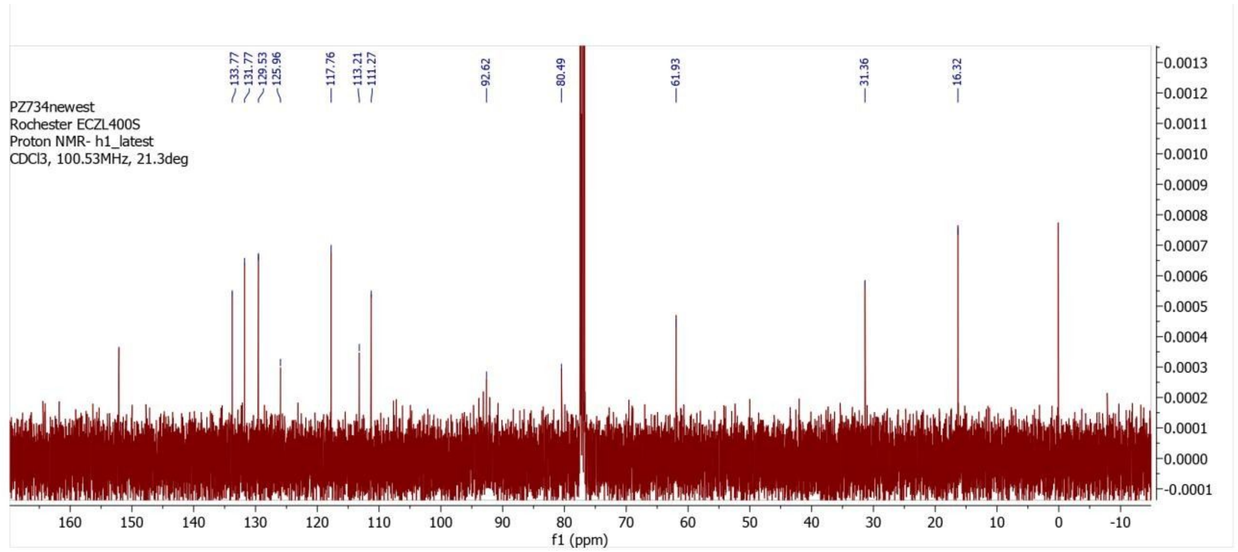
PZ734newest
Rochester ECZL400S
Proton NMR- h1_latest
CDCl3, 100.53MHz, 21.3deg
133.77
131.77
129.53
125.96
117.76
113.21
111.27
92.62
80.49
61.93
31.36
16.32
f1 (ppm)

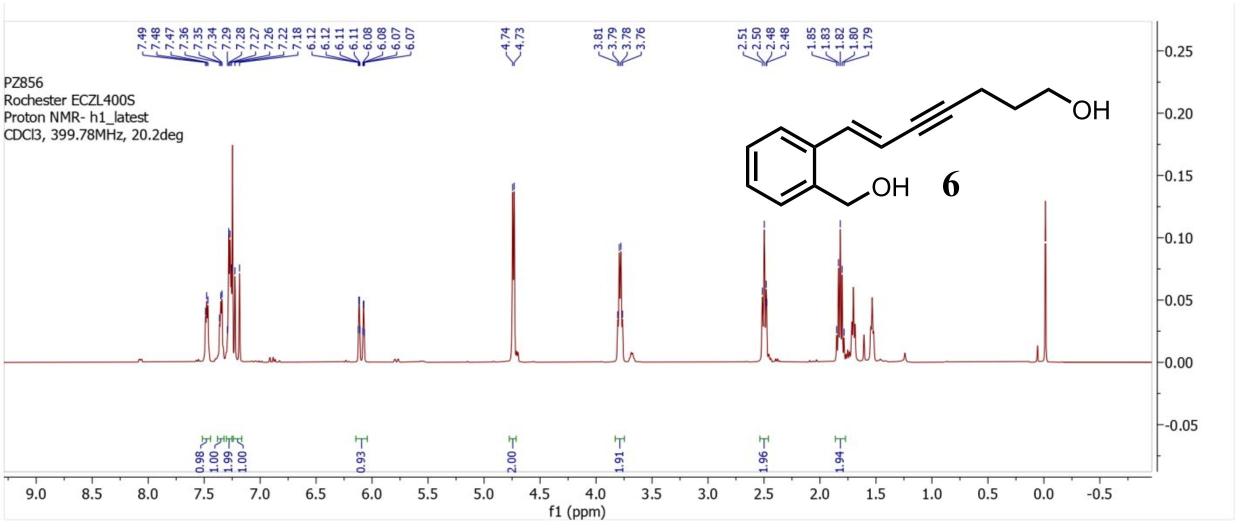
PZ856
Rochester ECZL400S
Proton NMR- h1_latest
CDCl3, 399.78MHz, 20.2deg
7.49 7.48 7.47 7.36 7.35 7.34 7.29 7.28 7.27 7.26 7.22 7.18 6.12 6.12 6.11 6.11 6.08 6.08 6.07 6.07
4.74 4.73
3.81 3.79 3.78 3.76
2.51 2.50 2.48 2.48
1.85 1.83 1.82 1.80 1.79
OH
OH
6
0.98 1.00 1.99 1.00
0.93
2.00
1.91
1.96
1.94
f1 (ppm)

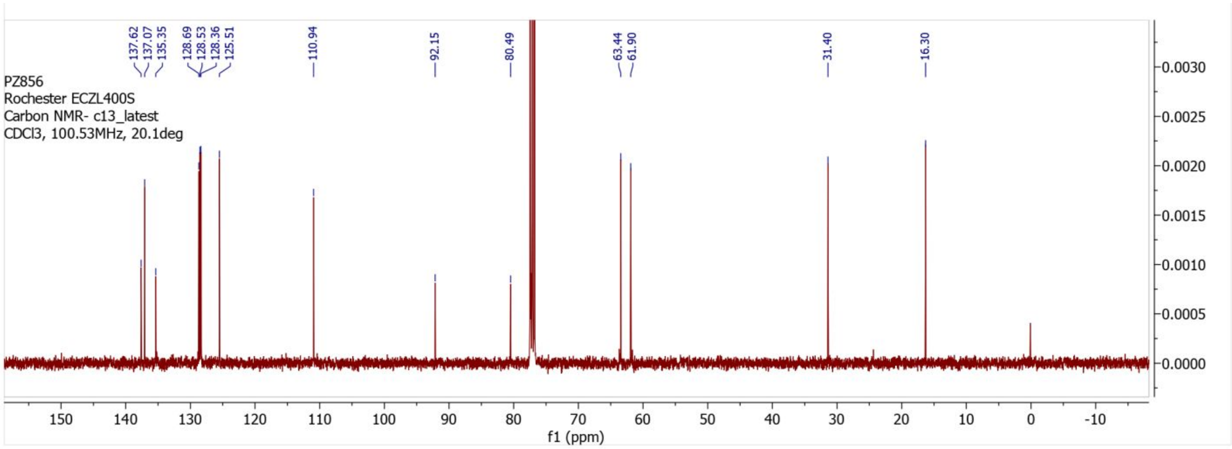
PZ856
Rochester ECZL400S
Carbon NMR- c13_latest
CDCl3, 100.53MHz, 20.1deg
137.62 137.07 135.35 128.69 128.53 128.36 125.51
110.94
92.15
80.49
63.44 61.90
31.40
16.30
f1 (ppm)

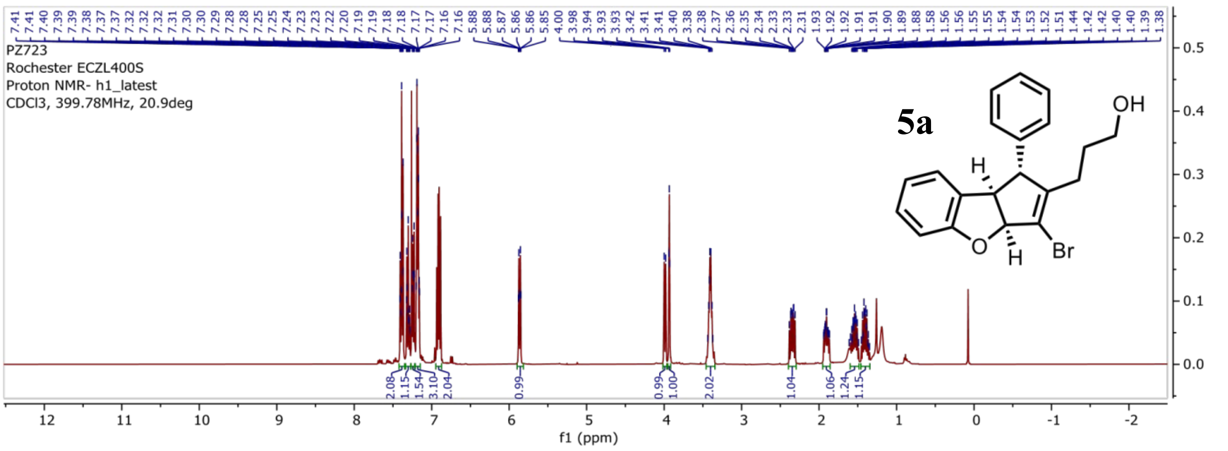
7.41 7.41 7.40 7.39 7.39 7.38 7.37 7.37 7.32 7.32 7.32 7.31 7.30 7.30 7.29 7.28 7.28 7.25 7.25 7.24 7.23 7.23 7.22 7.20 7.19 7.19 7.18 7.18 7.17 7.17 7.16 7.16 5.88 5.87 5.86 5.86 5.85 4.00 3.98 3.94 3.93 3.93 3.42 3.41 3.40 3.38 2.38 2.37 2.36 2.35 2.34 2.33 2.33 2.31 1.93 1.92 1.92 1.91 1.91 1.90 1.89 1.88 1.58 1.56 1.56 1.55 1.55 1.54 1.54 1.53 1.52 1.51 1.44 1.42 1.40 1.39 1.38
PZ723
Rochester ECZL400S
Proton NMR- h1_latest
CDCl3, 399.78MHz, 20.9deg
5a
OH
H
H
O
Br
2.08 1.15 1.54 3.10 2.04 0.99 0.99 1.00 2.02 1.04 1.06 1.24 1.15
f1 (ppm)

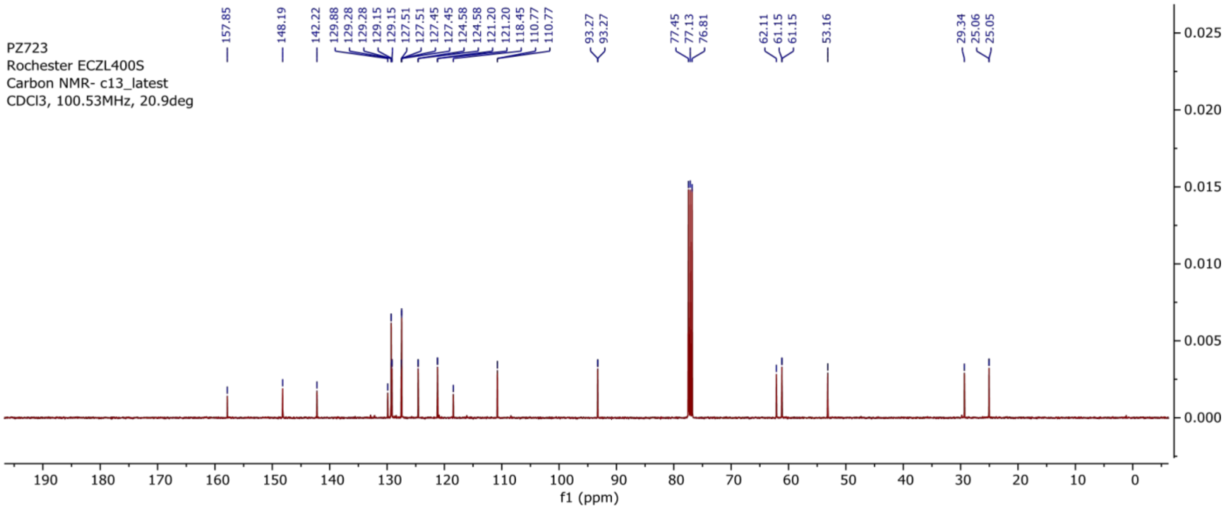
157.85 148.19 142.22 129.88 129.28 129.28 129.15 129.15 127.51 127.51 127.45 127.45 124.58 124.58 121.20 121.20 118.45 110.77 110.77 93.27 93.27 77.45 77.13 76.81 62.11 61.15 61.15 53.16 29.34 25.06 25.05
PZ723
Rochester ECZL400S
Carbon NMR- c13_latest
CDCl3, 100.53MHz, 20.9deg
f1 (ppm)

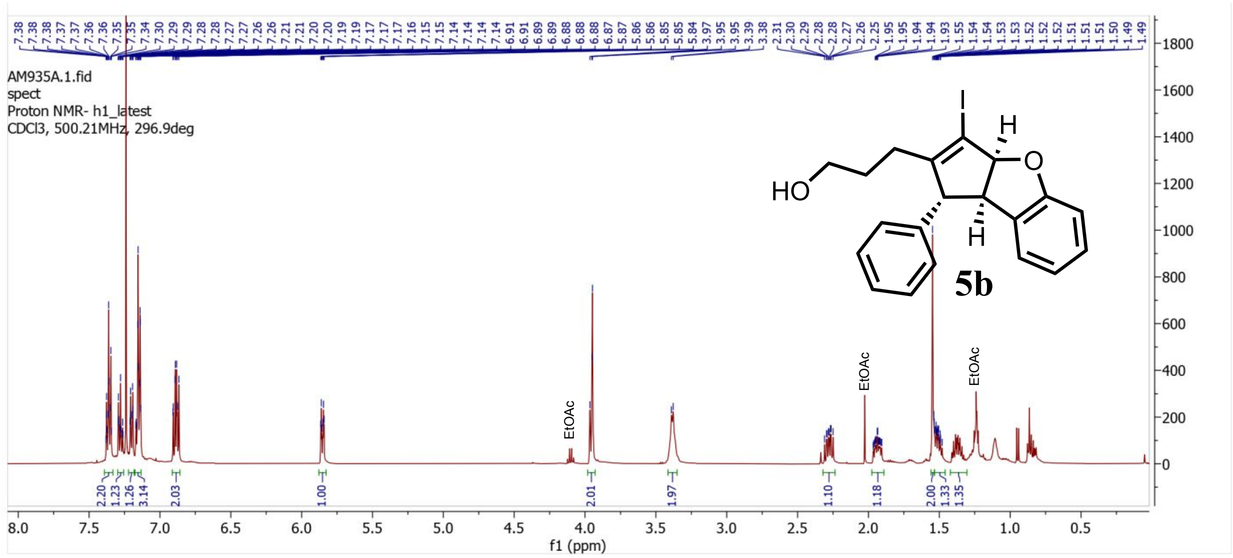
AM935A.1.fid
spect
Proton NMR- h1_latest
CDCl3, 500.21MHz, 296.9deg
HO
I
H
O
H
5b
EtOAc
EtOAc
EtOAc
f1 (ppm)

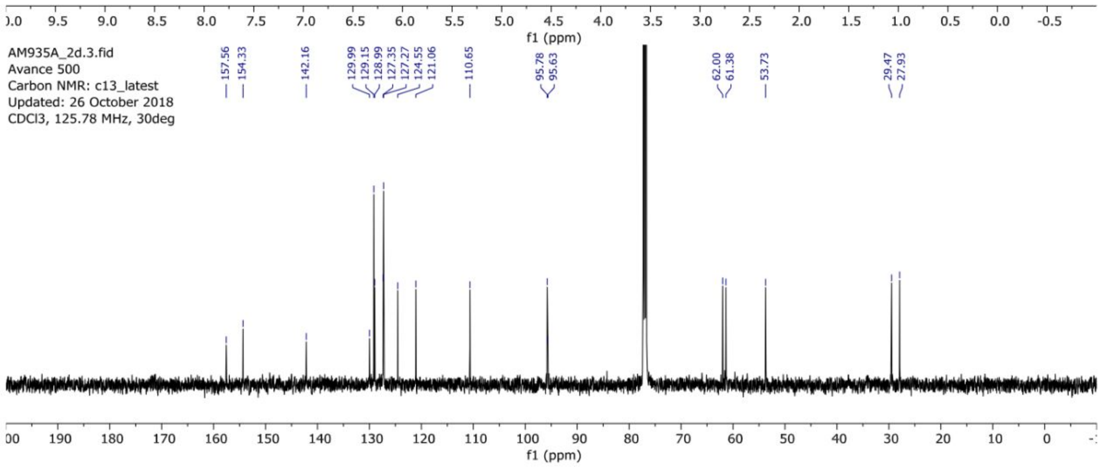
AM935A_2d.3.fid
Avance 500
Carbon NMR: c13_latest
Updated: 26 October 2018
CDCl3, 125.78 MHz, 30deg
157.56
154.33
142.16
129.99
129.15
128.99
127.35
127.27
124.55
121.06
110.65
95.78
95.63
62.00
61.38
53.73
29.47
27.93
f1 (ppm)

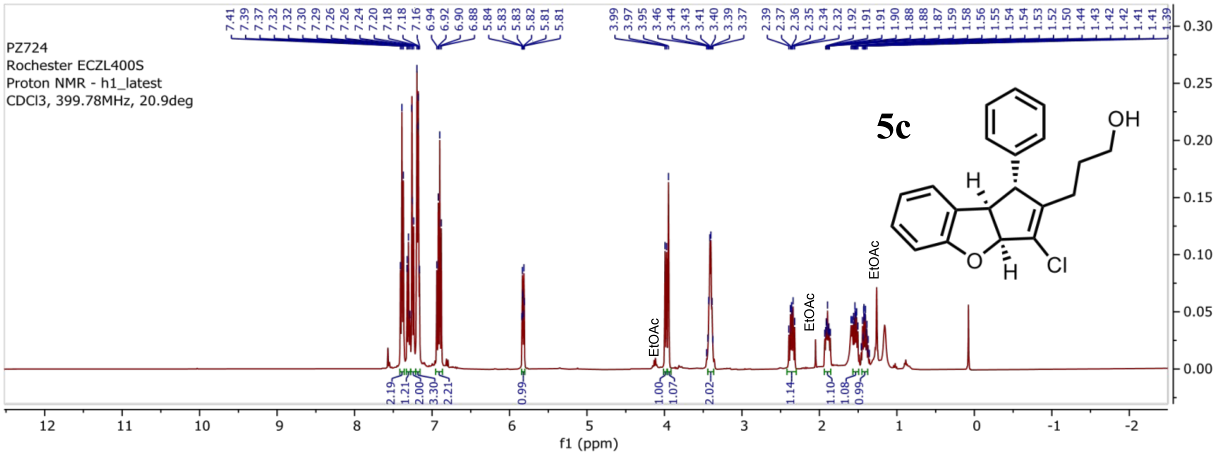
PZ724
Rochester ECZL400S
Proton NMR - h1_latest
CDCl3, 399.78MHz, 20.9deg
5c
OH
H
H
O
Cl
EtOAc
EtOAc
EtOAc
7.41 7.39 7.37 7.32 7.32 7.30 7.29 7.26 7.26 7.24 7.20 7.18 7.16 6.94 6.92 6.90 6.88 5.84 5.83 5.83 5.82 5.81 5.81
3.99 3.97 3.95 3.46 3.44 3.43 3.41 3.40 3.39 3.37
2.39 2.37 2.36 2.35 2.34 2.32 1.92 1.91 1.91 1.90 1.88 1.88 1.87 1.59 1.58 1.56 1.55 1.54 1.54 1.53 1.52 1.50 1.44 1.43 1.42 1.42 1.41 1.41 1.39
2.19 1.21 2.00 3.30 2.21 0.99 1.00 1.07 2.02 1.14 1.10 1.08 0.99
f1 (ppm)

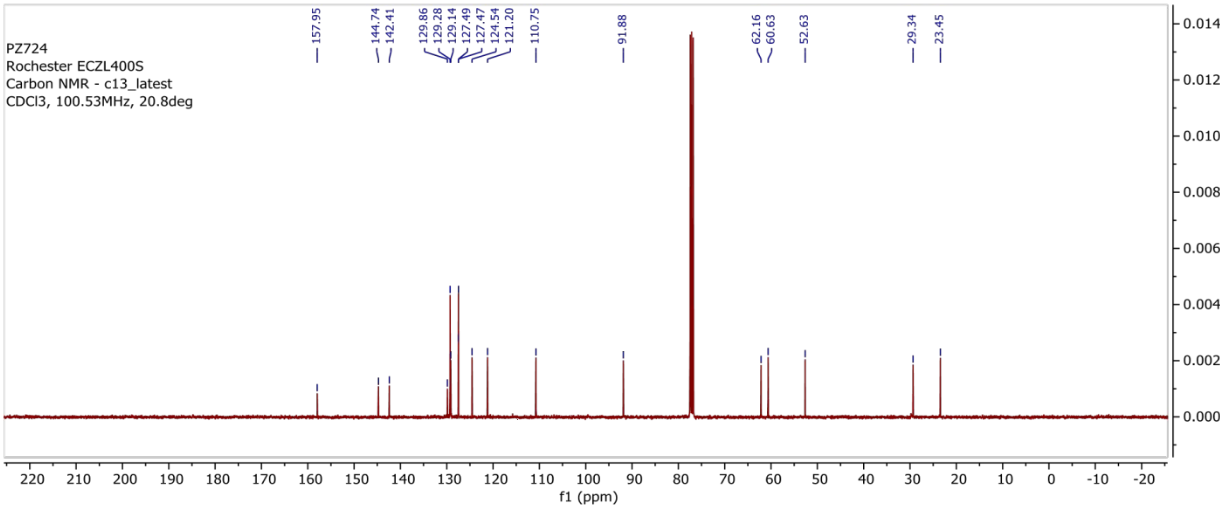
PZ724
Rochester ECZL400S
Carbon NMR - c13_latest
CDCl3, 100.53MHz, 20.8deg
157.95
144.74 142.41
129.86 129.28 129.14 127.49 127.47 124.54 121.20
110.75
91.88
62.16 60.63
52.63
29.34 23.45
f1 (ppm)

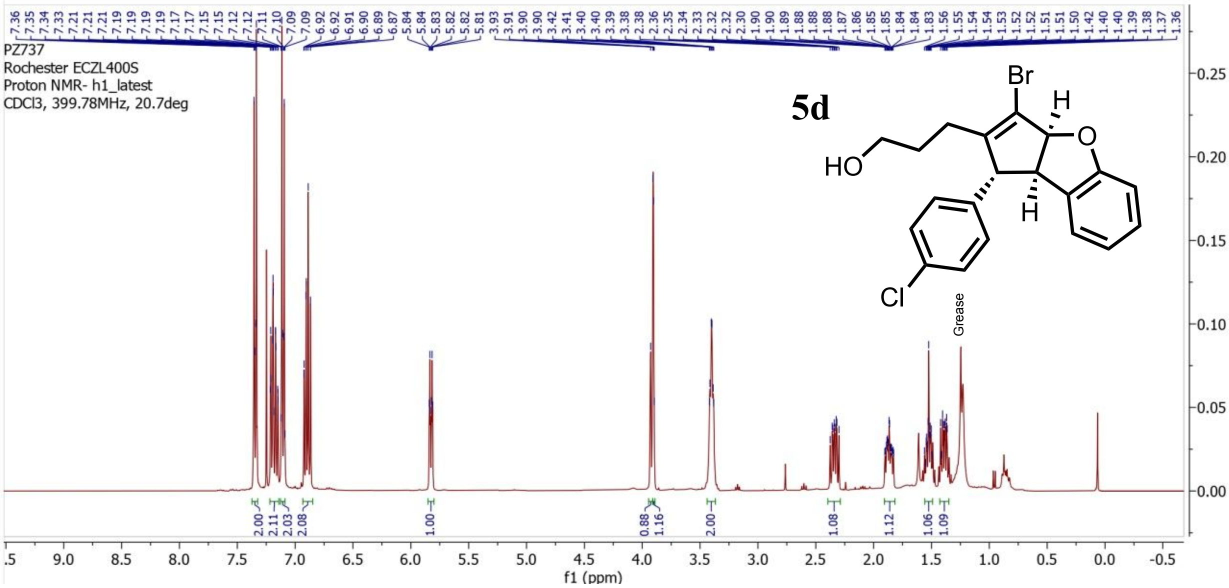
PZ737
Rochester ECZL400S
Proton NMR- h1_latest
CDCl3, 399.78MHz, 20.7deg
5d
Br
H
O
HO
H
Cl
Grease
f1 (ppm)

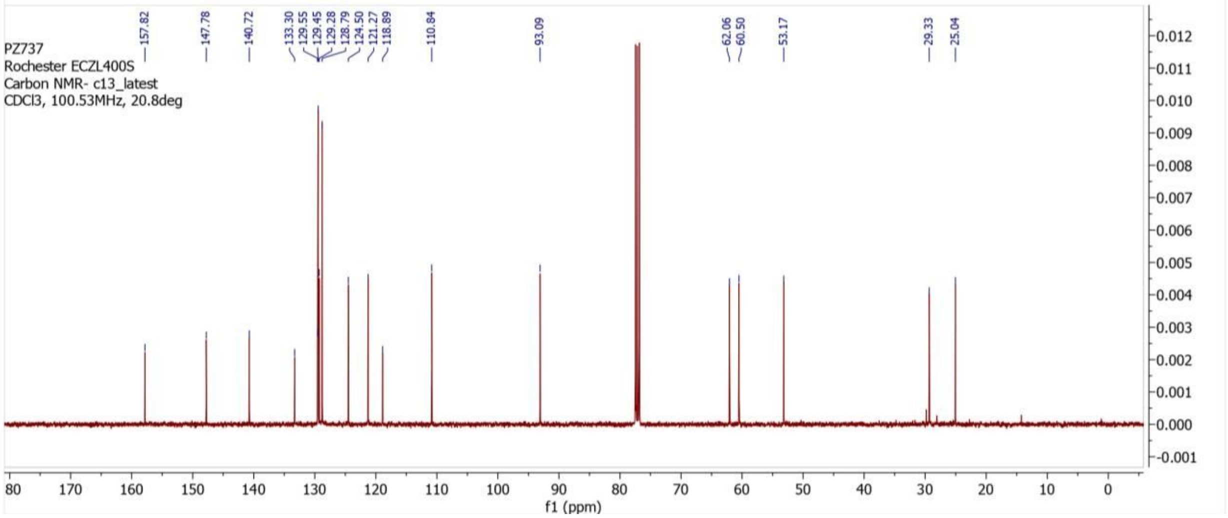
PZ737
Rochester ECZL400S
Carbon NMR- c13_latest
CDCl3, 100.53MHz, 20.8deg
157.82
147.78
140.72
133.30
129.55
129.45
129.28
128.79
124.50
121.27
118.89
110.84
93.09
62.06
60.50
53.17
29.33
25.04
f1 (ppm)

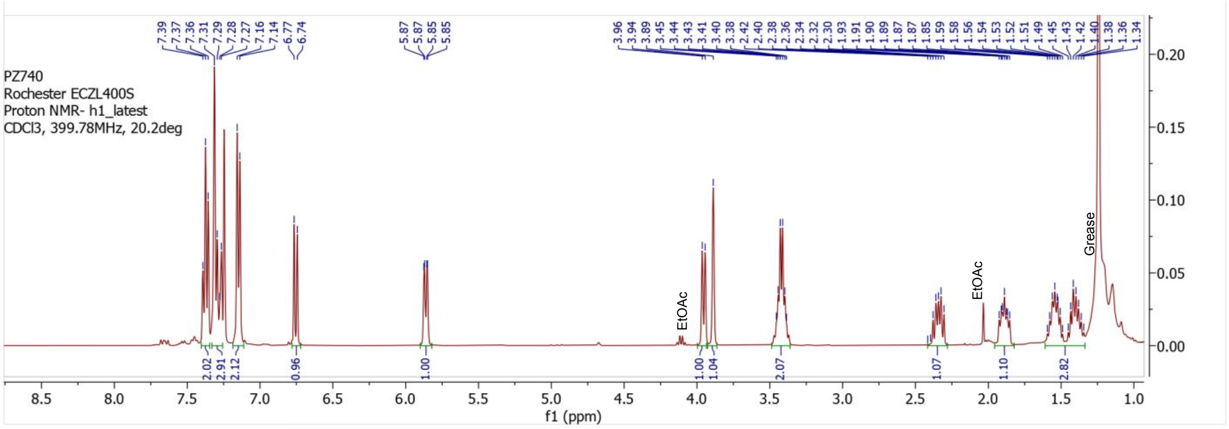
PZ740
Rochester ECZL400S
Proton NMR- h1_latest
CDCl3, 399.78MHz, 20.2deg
EtOAc
EtOAc
Grease
f1 (ppm)

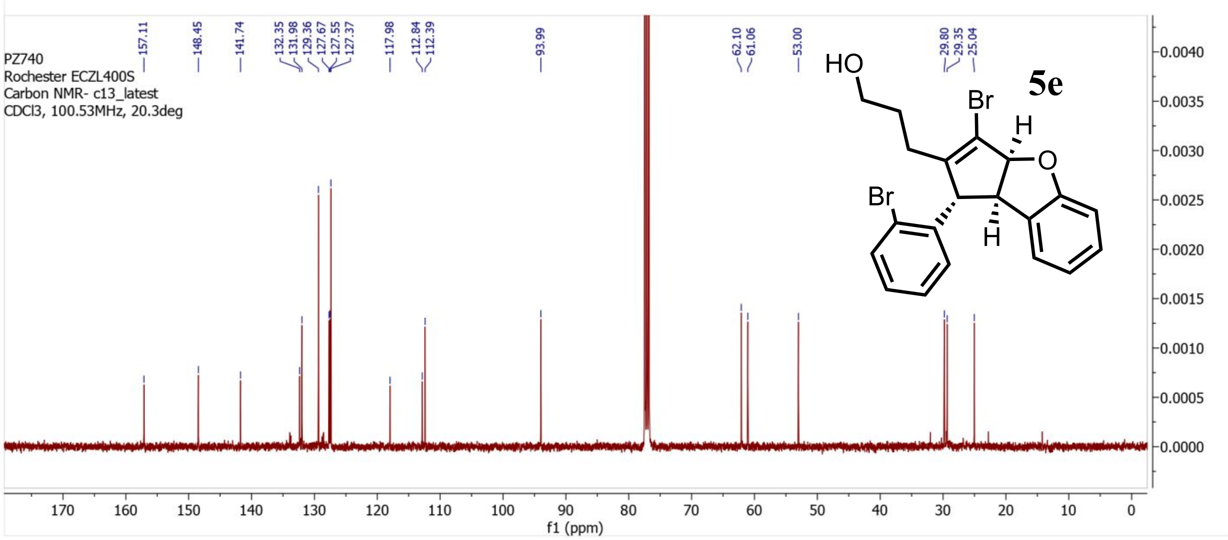
PZ740
Rochester ECZL400S
Carbon NMR- c13_latest
CDCl3, 100.53MHz, 20.3deg
HO
Br
Br
H
H
O
5e
f1 (ppm)

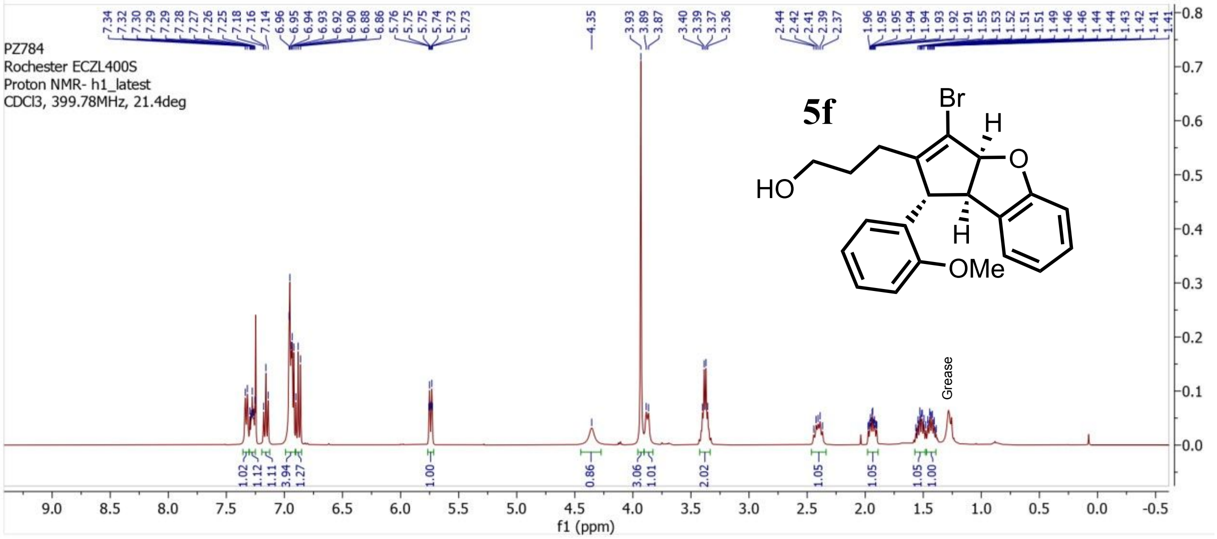
PZ784
Rochester ECZL400S
Proton NMR- h1_latest
CDCl3, 399.78MHz, 21.4deg
5f
Br
H
O
HO
H
OMe
Grease
f1 (ppm)

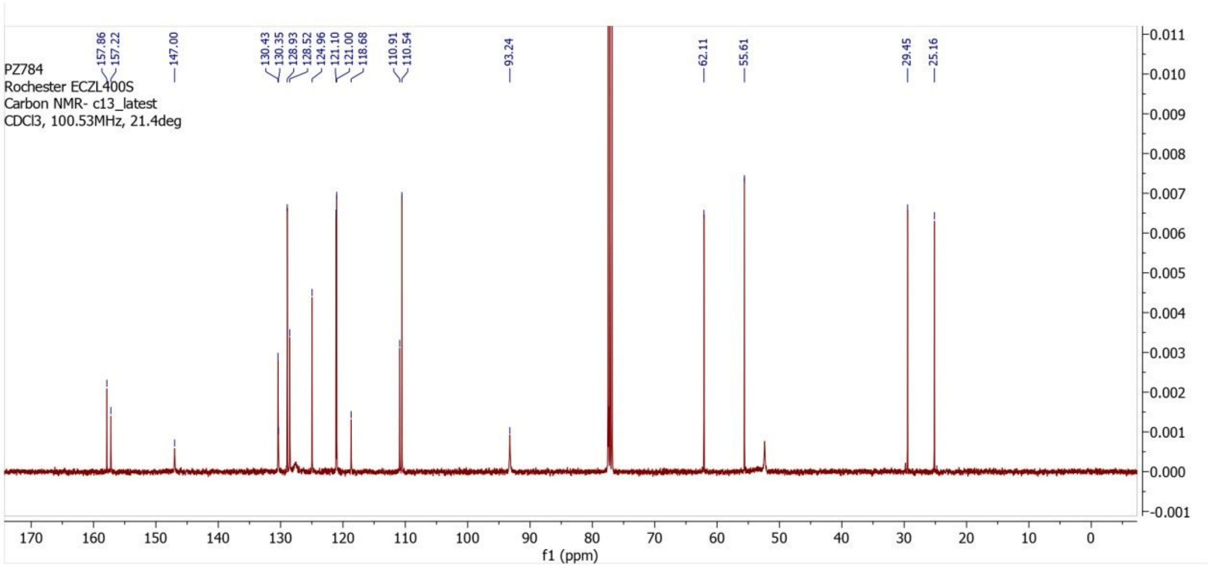
PZ784
Rochester ECZL400S
Carbon NMR- c13_latest
CDCl3, 100.53MHz, 21.4deg
f1 (ppm)

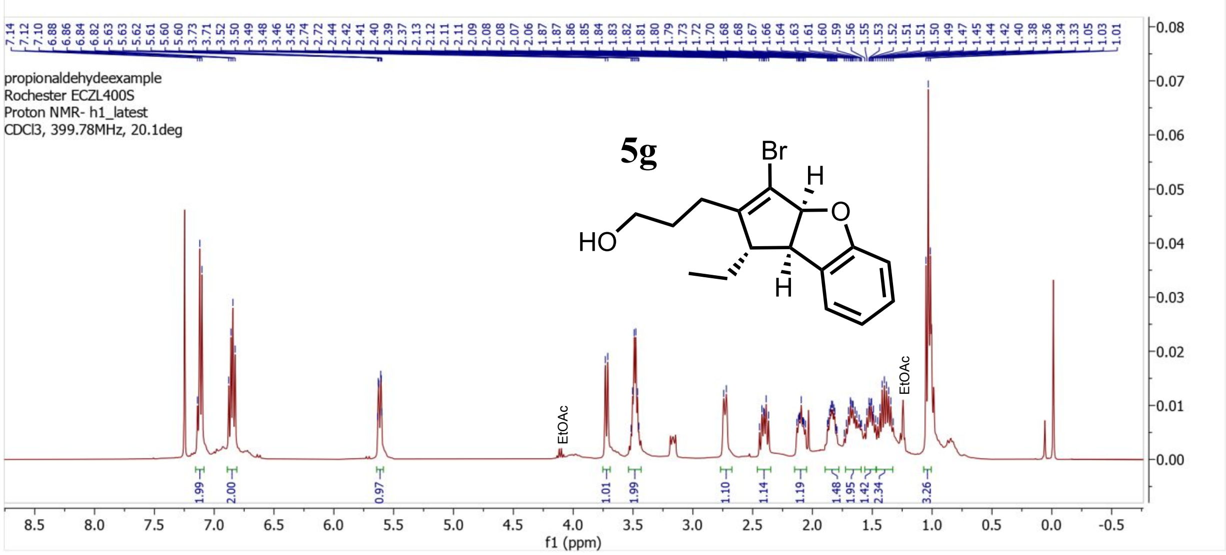
propionaldehydeexample
Rochester ECZL400S
Proton NMR- h1_latest
CDCl3, 399.78MHz, 20.1deg
5g
Br
H
O
HO
H
EtOAc
EtOAc
f1 (ppm)

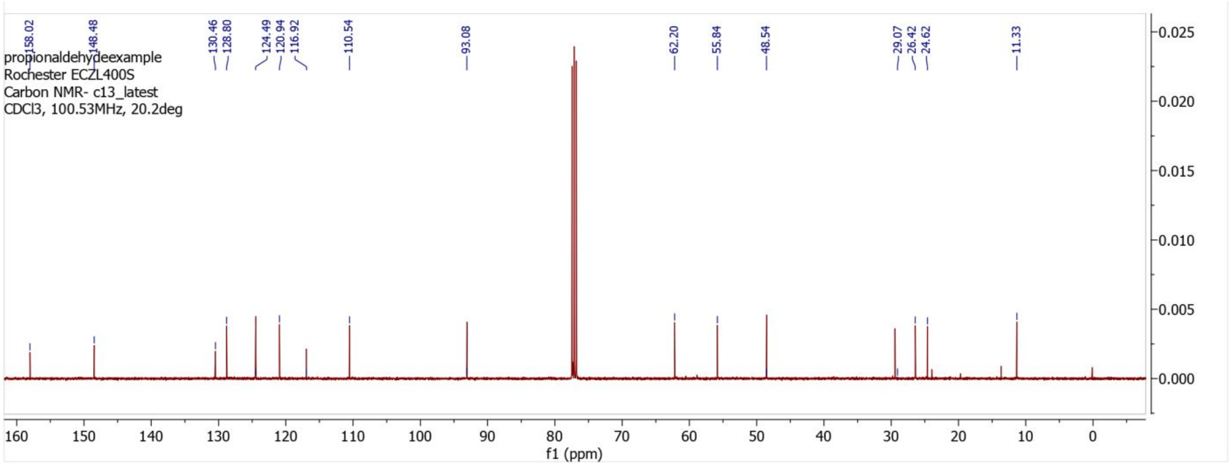
propionaldehydeexample
Rochester ECZL400S
Carbon NMR- c13_latest
CDCl3, 100.53MHz, 20.2deg
158.02
148.48
130.46
128.80
124.49
120.94
116.92
110.54
93.08
62.20
55.84
48.54
29.07
26.42
24.62
11.33
f1 (ppm)

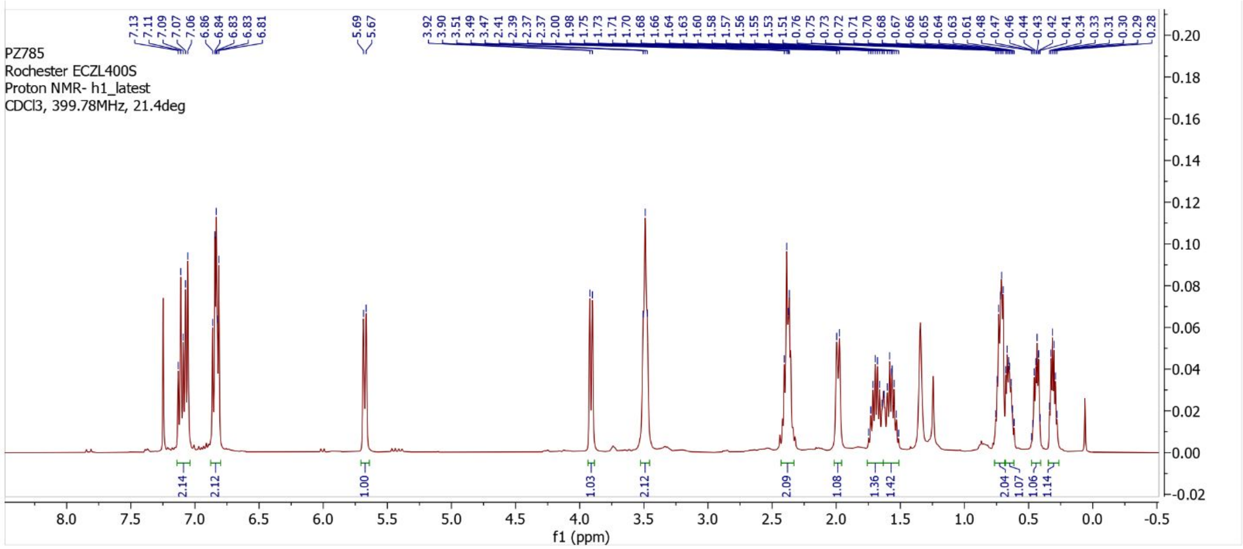
PZ785
Rochester ECZL400S
Proton NMR- h1_latest
CDCl3, 399.78MHz, 21.4deg
f1 (ppm)

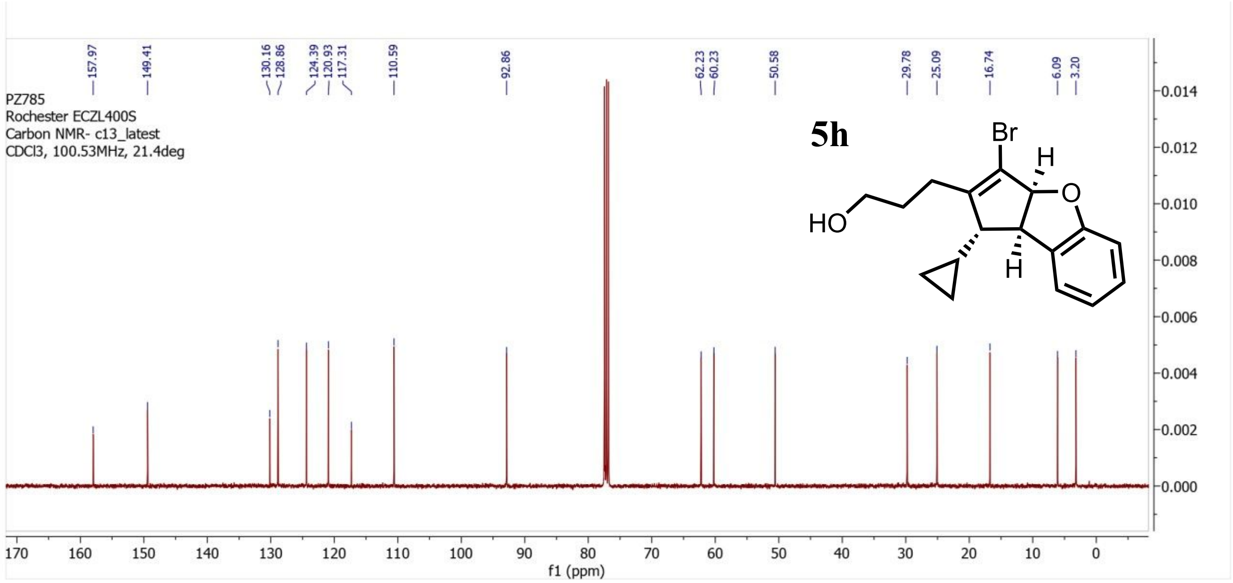
PZ785
Rochester ECZL400S
Carbon NMR- c13_latest
CDCl3, 100.53MHz, 21.4deg
5h
Br
H
O
HO
H
f1 (ppm)

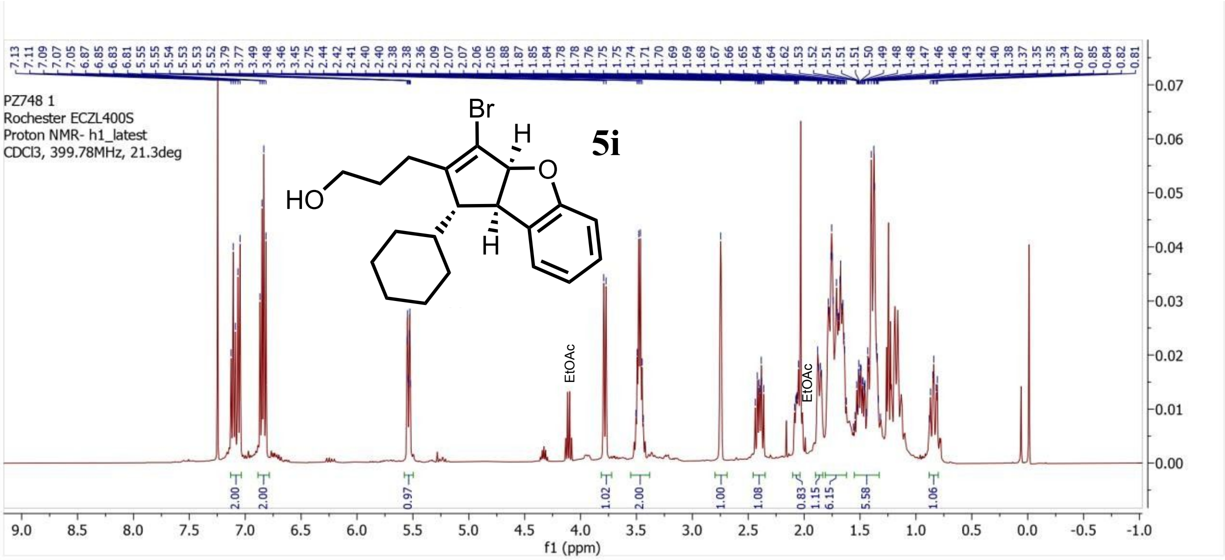
PZ748 1
Rochester ECZL400S
Proton NMR- h1_latest
CDCl3, 399.78MHz, 21.3deg
5i
Br
H
O
HO
H
EtOAc
EtOAc
f1 (ppm)

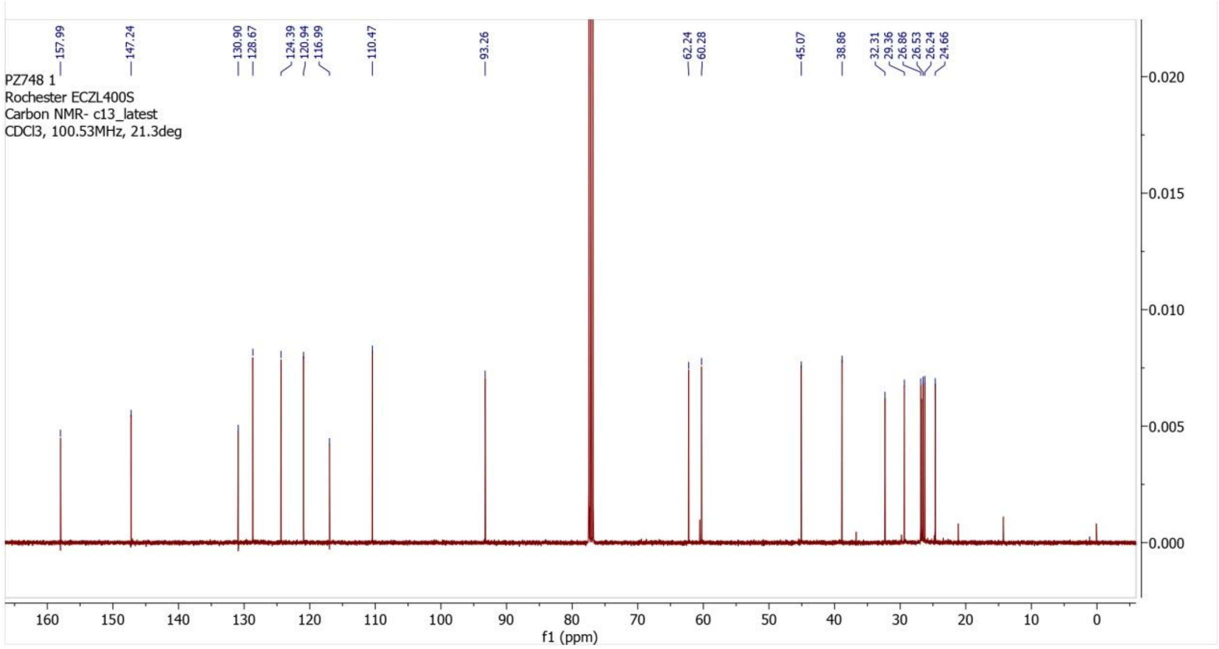
PZ748 1
Rochester ECZL400S
Carbon NMR- c13_latest
CDCl3, 100.53MHz, 21.3deg
157.99
147.24
130.90
128.67
124.39
120.94
116.99
110.47
93.26
62.24
60.28
45.07
38.86
32.31
29.36
26.86
26.53
26.24
24.66
f1 (ppm)

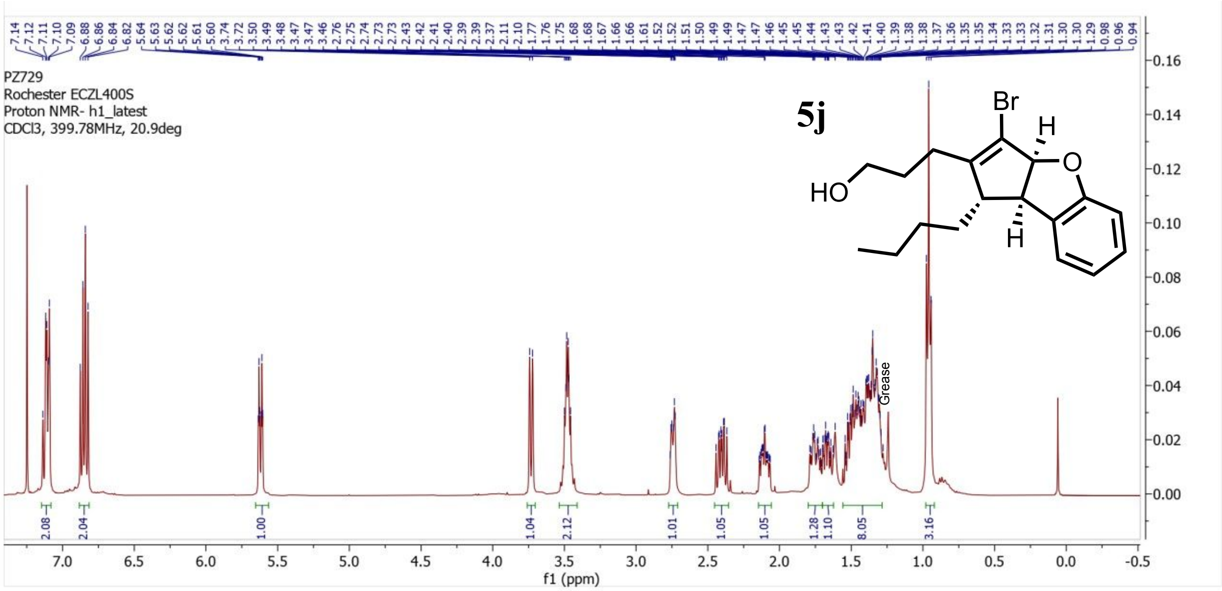
PZ729
Rochester ECZL400S
Proton NMR- h1_latest
CDCl3, 399.78MHz, 20.9deg
5j
Br
H
HO
O
H
Grease
f1 (ppm)

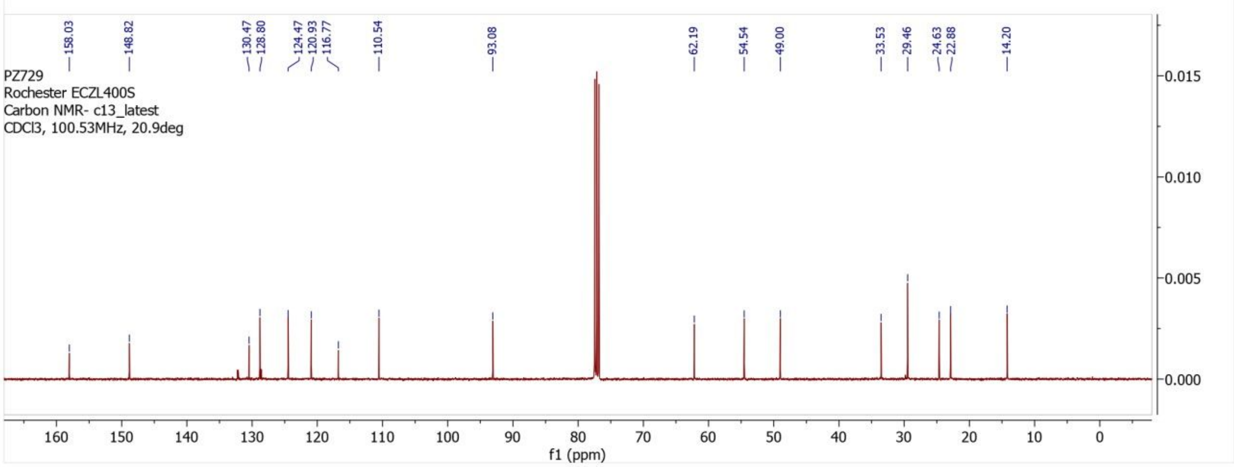
PZ729
Rochester ECZL400S
Carbon NMR- c13_latest
CDCl3, 100.53MHz, 20.9deg
158.03
148.82
130.47
128.80
124.47
120.93
116.77
110.54
93.08
62.19
54.54
49.00
33.53
29.46
24.63
22.88
14.20
f1 (ppm)

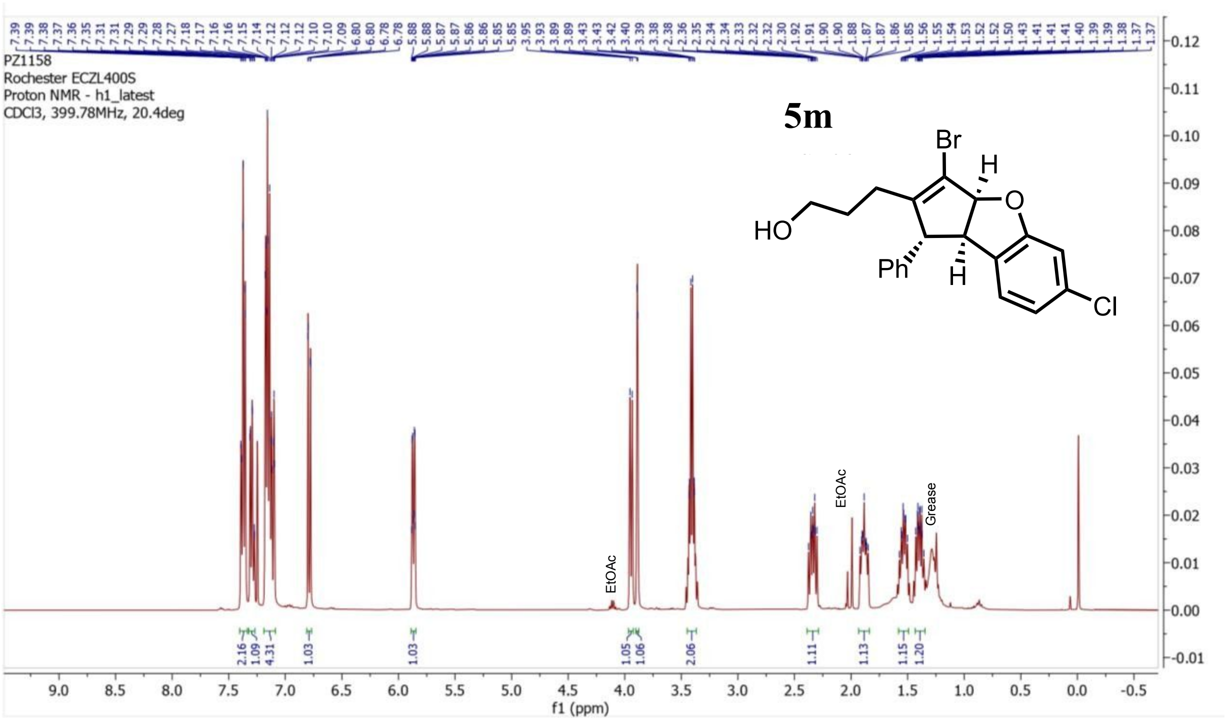
PZ1158
Rochester ECZL400S
Proton NMR - h1_latest
CDCl3, 399.78MHz, 20.4deg
5m
Br
H
O
HO
Ph
H
Cl
EtOAc
EtOAc
Grease
2.16
1.09
4.31
1.03
1.03
1.05
1.06
2.06
1.11
1.13
1.15
1.20
f1 (ppm)

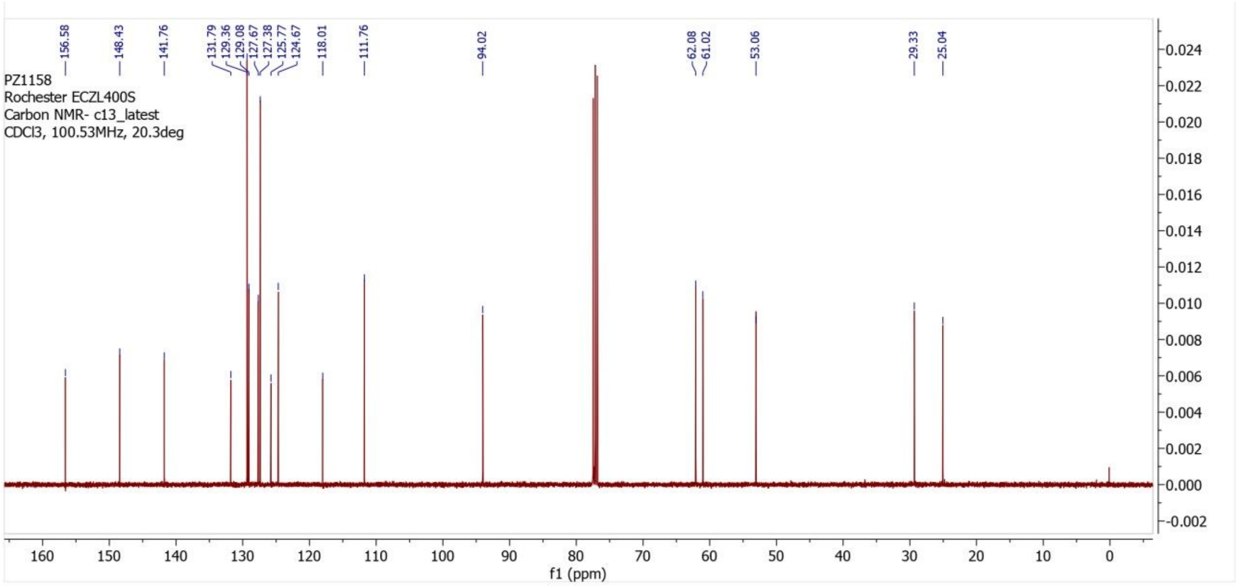
156.58
148.43
141.76
131.79
129.36
129.08
127.67
127.38
125.77
124.67
118.01
111.76
94.02
62.08
61.02
53.06
29.33
25.04
PZ1158
Rochester ECZL400S
Carbon NMR- c13_latest
CDCl3, 100.53MHz, 20.3deg
f1 (ppm)

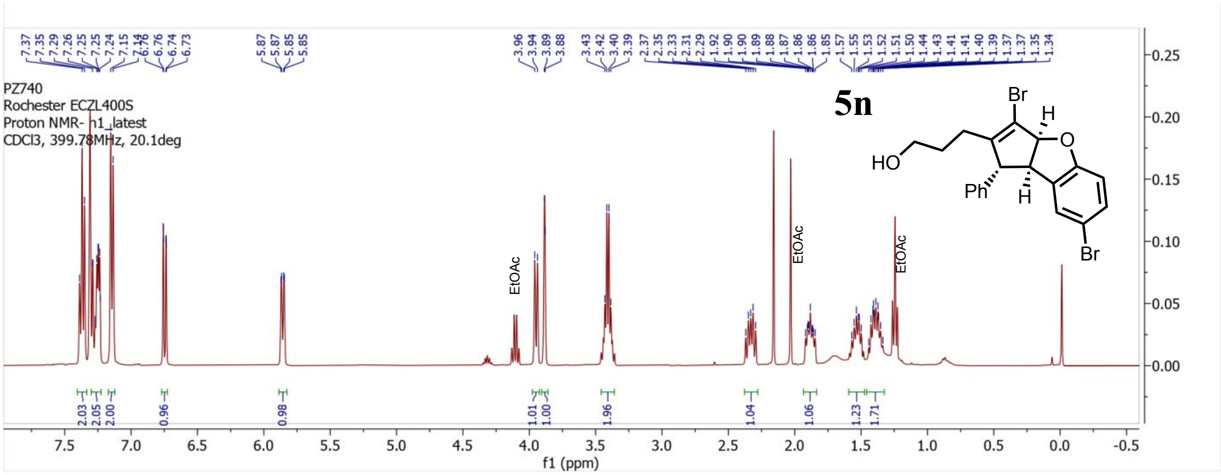
PZ740
Rochester ECZL400S
Proton NMR- h1_latest
CDCl3, 399.78MHz, 20.1deg
5n
EtOAc
f1 (ppm)

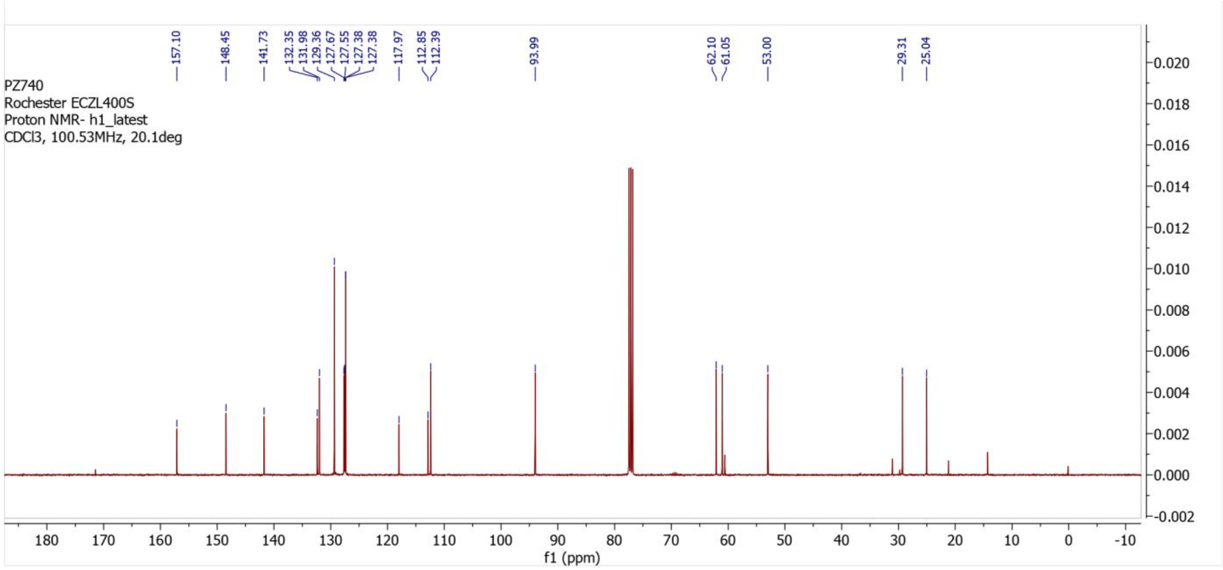
PZ740
Rochester ECZL400S
Proton NMR- h1_latest
CDCl3, 100.53MHz, 20.1deg
f1 (ppm)

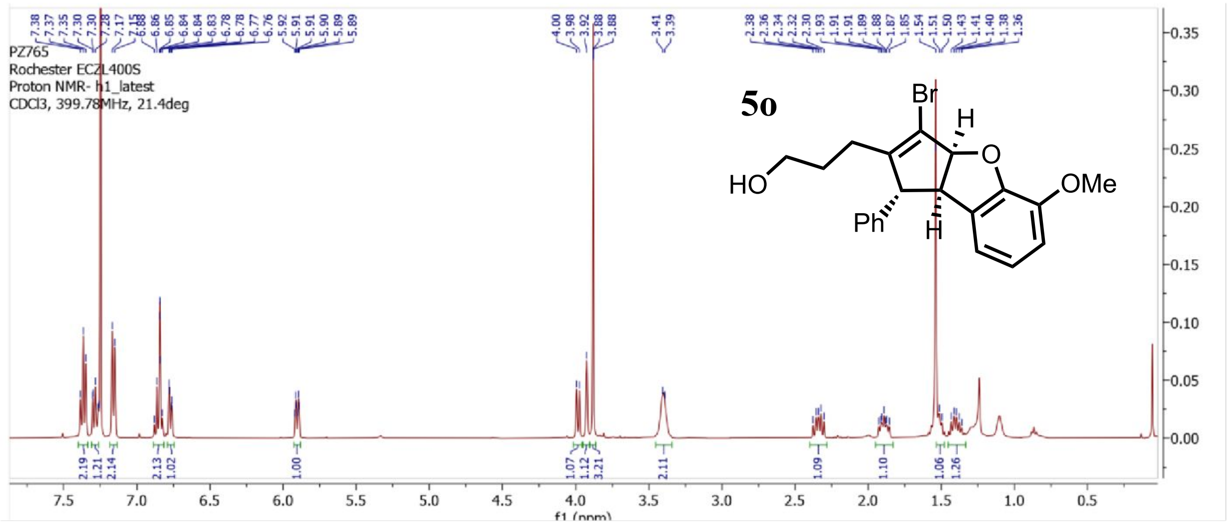
PZ765
Rochester ECZL400S
Proton NMR- h1_latest
CDCl3, 399.78MHz, 21.4deg
5o
Br
H
O
HO
OMe
Ph
H
f1 (ppm)

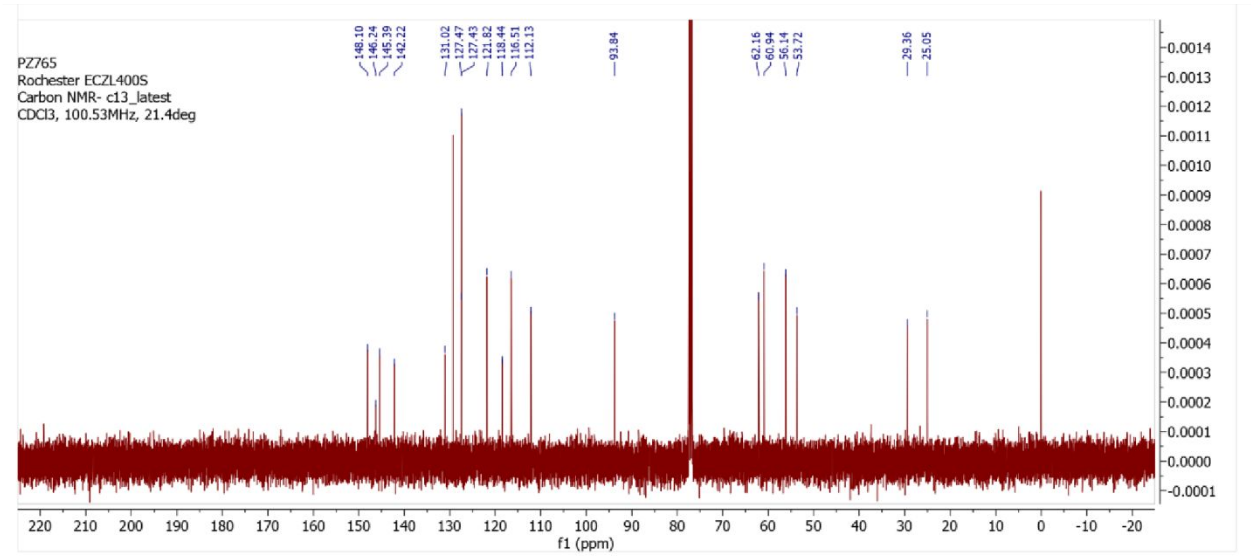
PZ765
Rochester ECZL400S
Carbon NMR- c13_latest
CDCl3, 100.53MHz, 21.4deg
148.10
146.24
145.39
142.22
131.02
127.47
127.43
121.82
118.44
116.51
112.13
93.84
62.16
60.94
56.14
53.72
29.36
25.05
f1 (ppm)

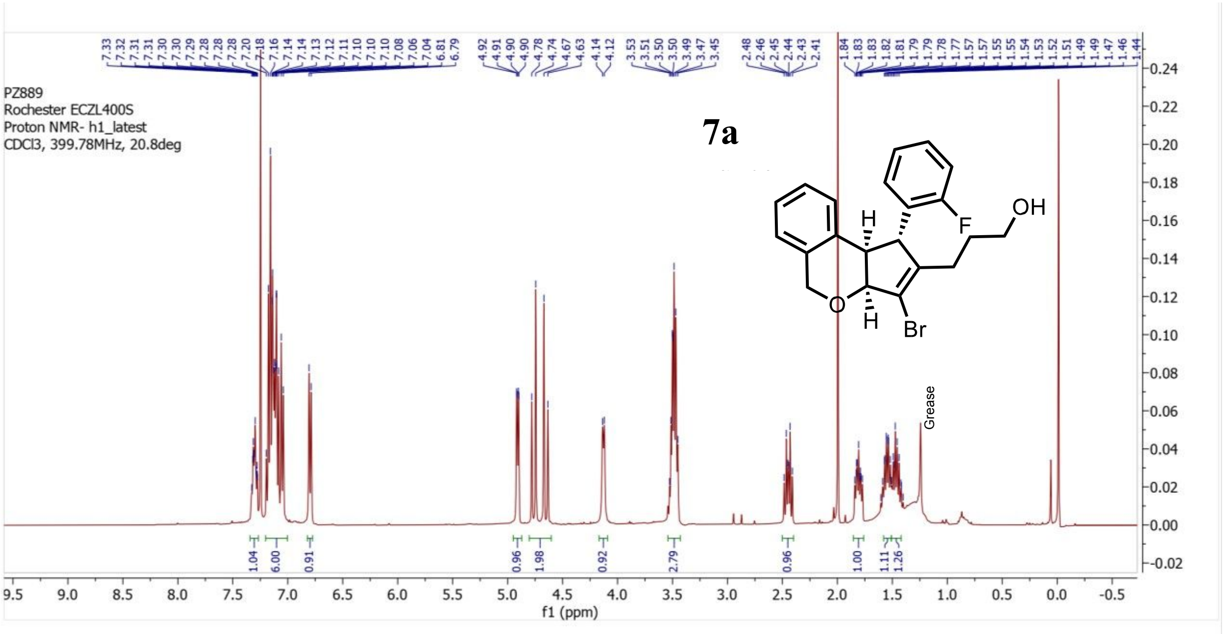
PZ889
Rochester ECZL400S
Proton NMR- h1_latest
CDCl3, 399.78MHz, 20.8deg
7a
OH
F
H
H
O
Br
Grease
f1 (ppm)

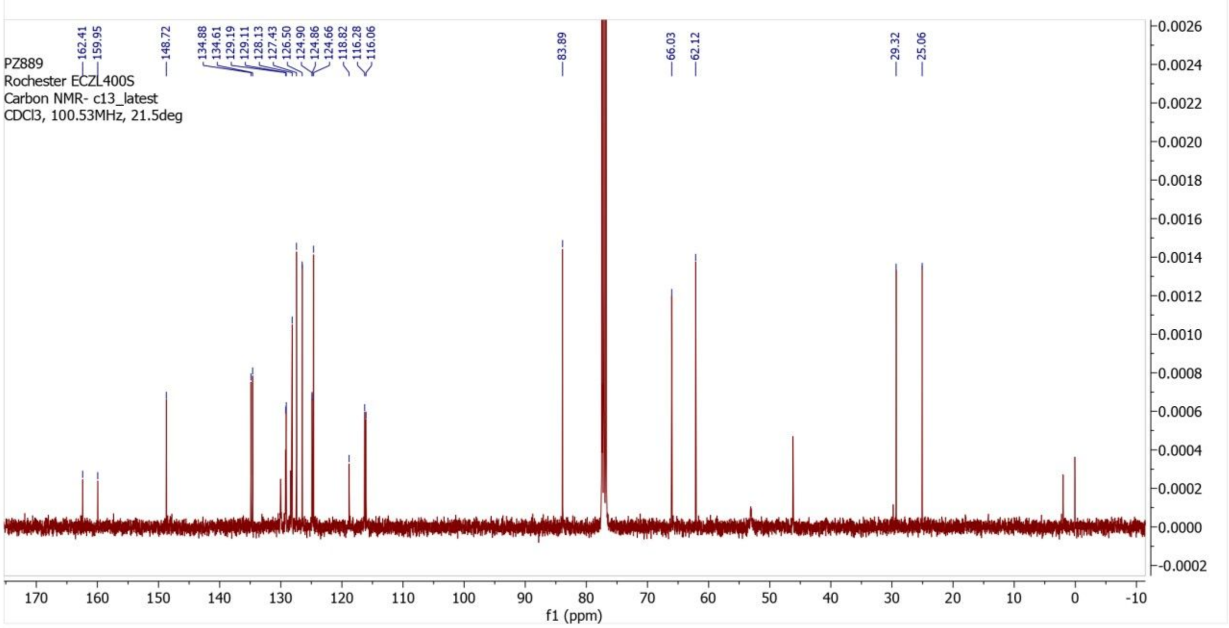
PZ889
Rochester ECZL400S
Carbon NMR- c13_latest
CDCl3, 100.53MHz, 21.5deg
162.41
159.95
148.72
134.88
134.61
129.19
129.11
128.13
127.43
126.50
124.90
124.86
124.66
118.82
116.28
116.06
83.89
66.03
62.12
29.32
25.06
f1 (ppm)

7b

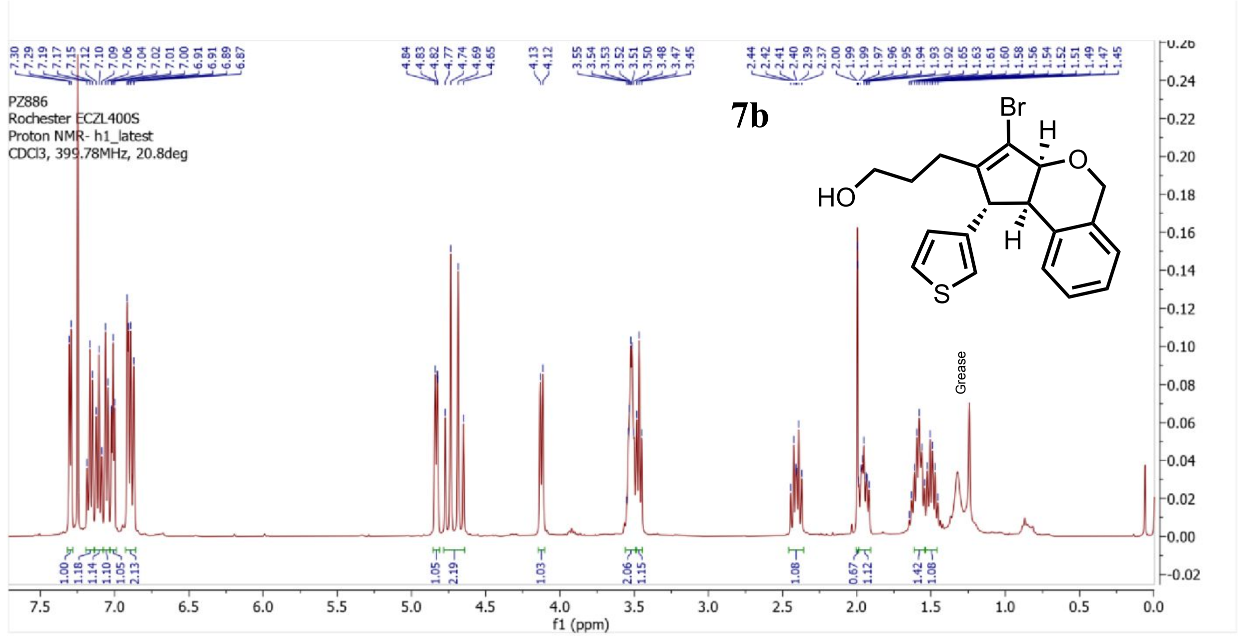
PZ886
Rochester ECZL400S
Proton NMR- h1_latest
CDCl3, 399.78MHz, 20.8deg
Br
H
O
HO
H
S
Grease
f1 (ppm)

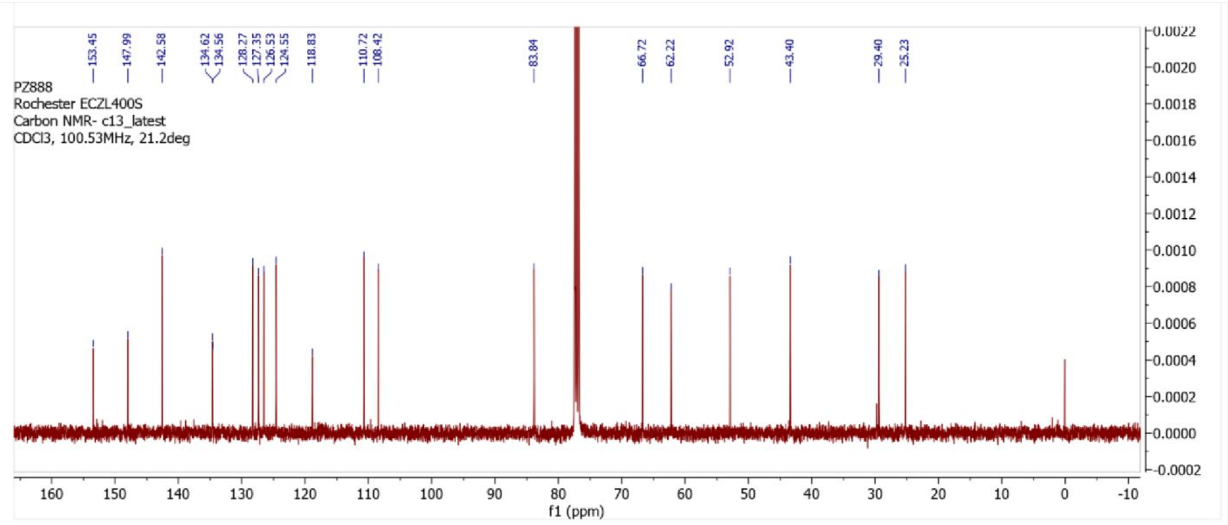
PZ888
Rochester ECZL400S
Carbon NMR- c13_latest
CDCl3, 100.53MHz, 21.2deg
153.45
147.99
142.58
134.62
134.56
128.27
127.35
126.53
124.55
118.83
110.72
108.42
83.84
66.72
62.22
52.92
43.40
29.40
25.23
f1 (ppm)

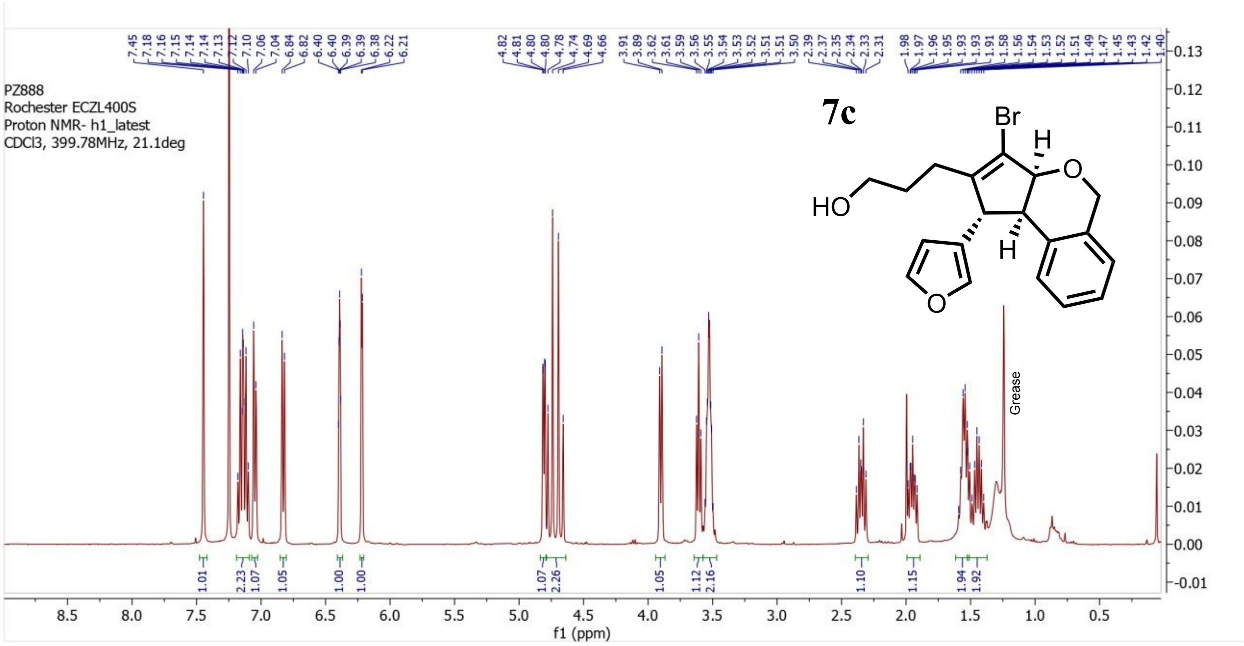

7.45 7.18 7.16 7.15 7.14 7.14 7.13 7.12 7.10 7.06 7.04 6.84 6.82 6.40 6.39 6.39 6.38 6.22 6.21
4.82 4.81 4.80 4.80 4.78 4.74 4.69 4.66
3.91 3.89 3.62 3.61 3.59 3.56 3.55 3.54 3.53 3.52 3.51 3.51 3.50
2.39 2.37 2.35 2.34 2.33 2.31
1.98 1.97 1.96 1.95 1.93 1.93 1.91 1.58 1.56 1.54 1.53 1.52 1.51 1.49 1.47 1.45 1.43 1.42 1.40
PZ888
Rochester ECZL400S
Proton NMR- h1_latest
CDCl3, 399.78MHz, 21.1deg
7c
Br
H
O
HO
H
O
Grease
1.01 2.23 1.07 1.05 1.00 1.00 1.07 2.26 1.05 1.12 2.16 1.10 1.15 1.94 1.92
f1 (ppm)

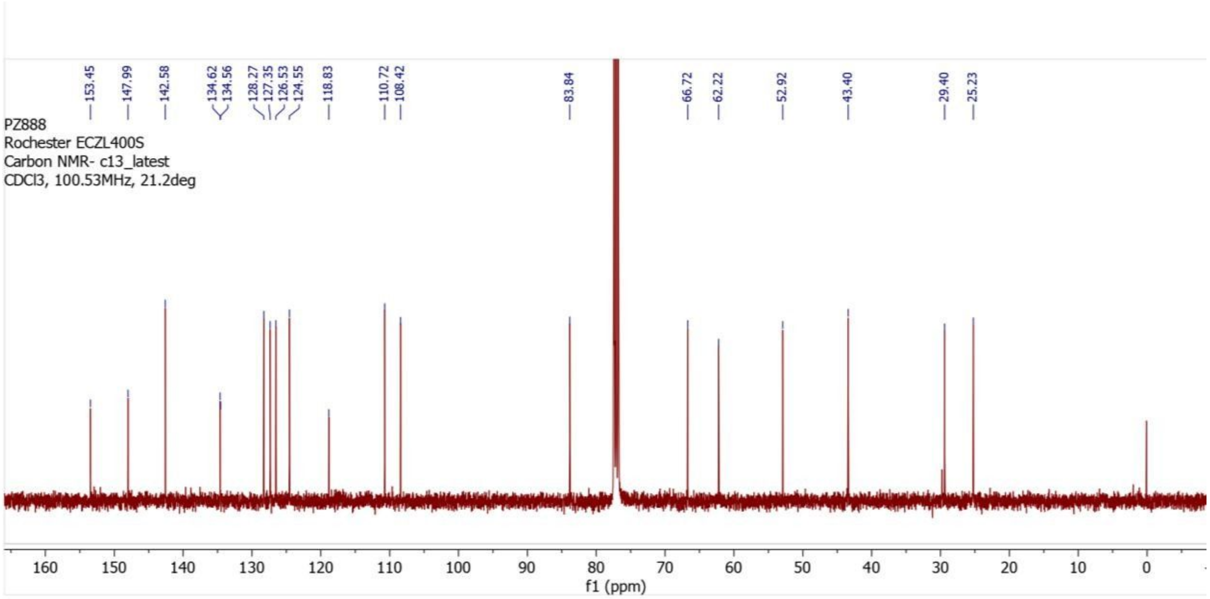

153.45 147.99 142.58 134.62 134.56 128.27 127.35 126.53 124.55 118.83 110.72 108.42 83.84 66.72 62.22 52.92 43.40 29.40 25.23
PZ888
Rochester ECZL400S
Carbon NMR- c13_latest
CDCl3, 100.53MHz, 21.2deg
f1 (ppm)

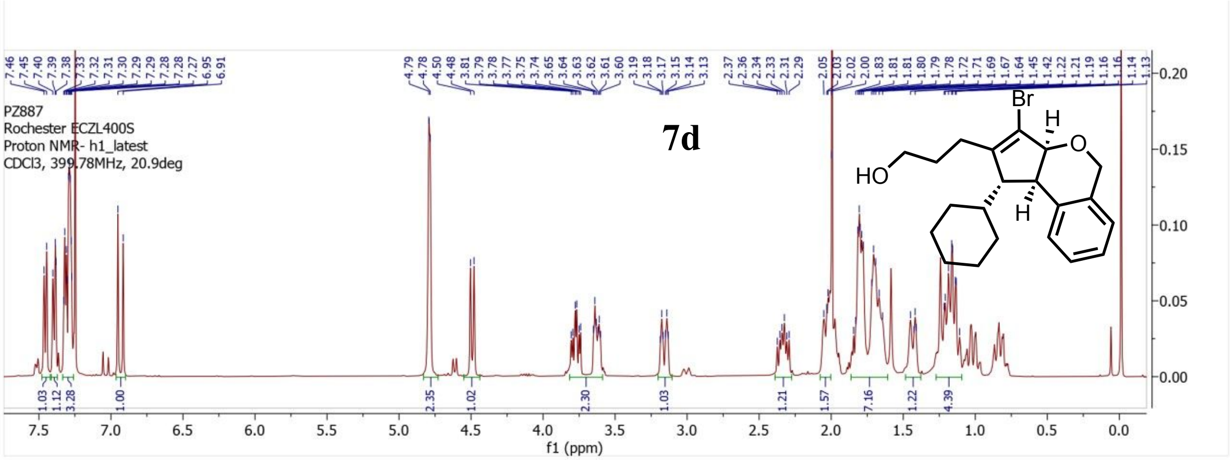

PZ887
Rochester ECZL400S
Proton NMR- h1_latest
CDCl3, 399.78MHz, 20.9deg
7d
Br
H
O
HO
H
f1 (ppm)

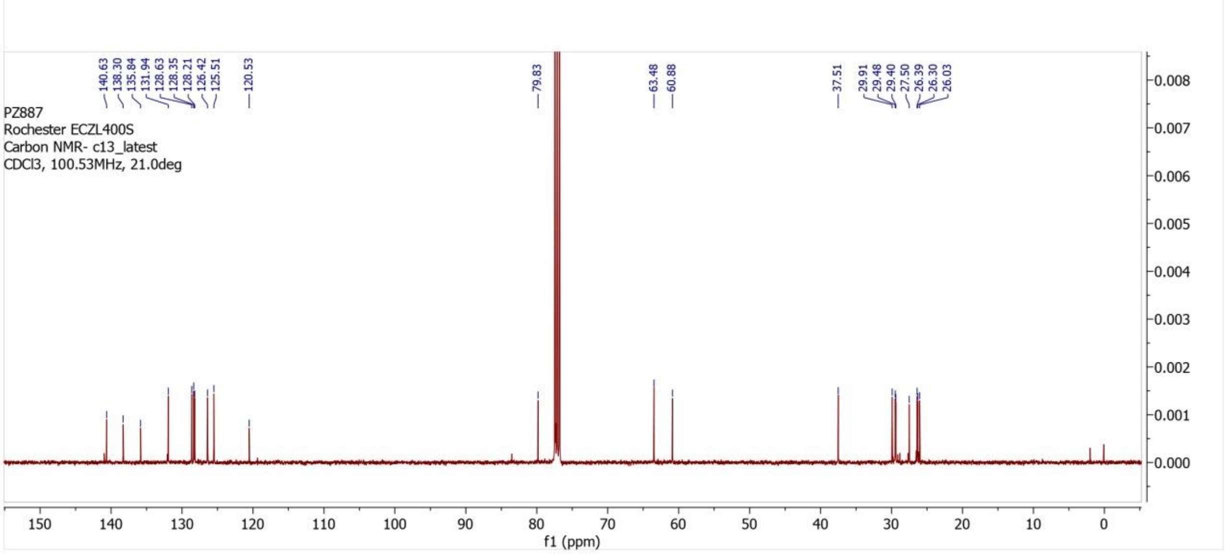

PZ887
Rochester ECZL400S
Carbon NMR- c13_latest
CDCl3, 100.53MHz, 21.0deg
140.63
138.30
135.84
131.94
128.63
128.35
128.21
126.42
125.51
120.53
79.83
63.48
60.88
37.51
29.91
29.48
29.40
27.50
26.39
26.30
26.03
f1 (ppm)

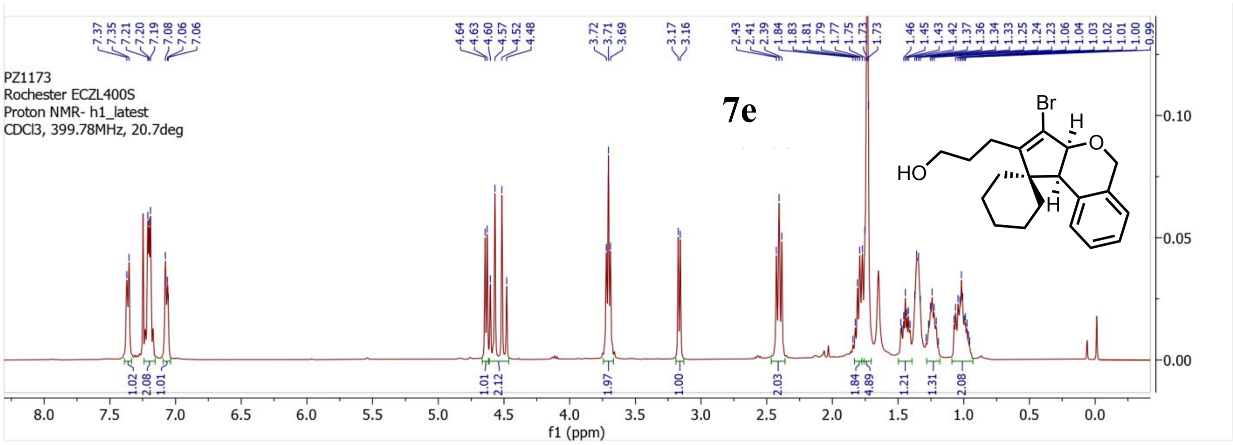
PZ1173
Rochester ECZL400S
Proton NMR- h1_latest
CDCl3, 399.78MHz, 20.7deg
7e
Br
H
O
HO
H
f1 (ppm)

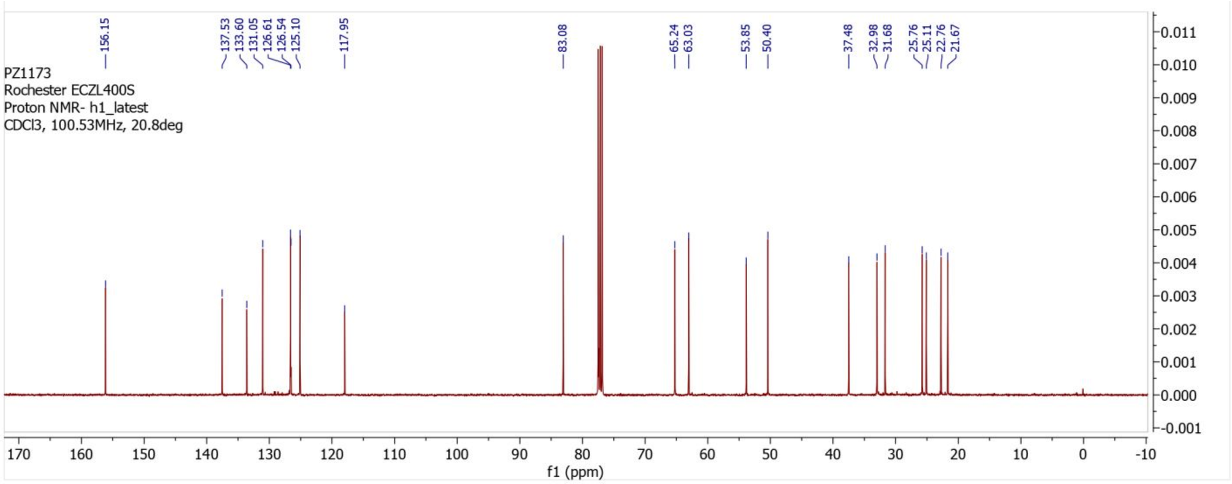
PZ1173
Rochester ECZL400S
Proton NMR- h1_latest
CDCl3, 100.53MHz, 20.8deg
156.15
137.53
133.60
131.05
126.61
126.54
125.10
117.95
83.08
65.24
63.03
53.85
50.40
37.48
32.98
31.68
25.76
25.11
22.76
21.67
f1 (ppm)

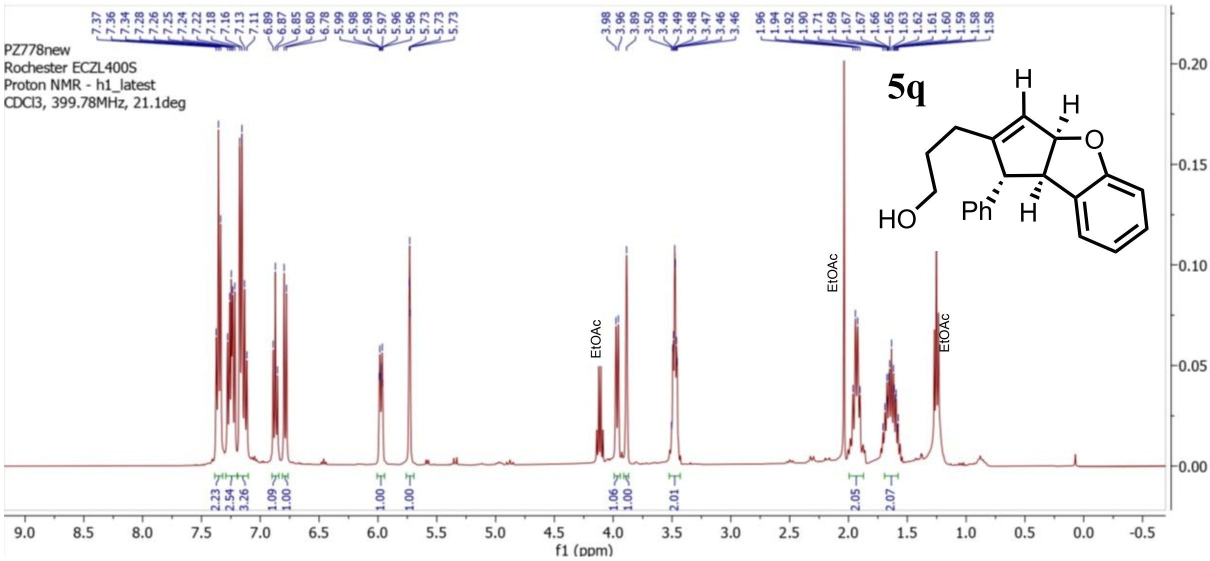
PZ778new
Rochester ECZL400S
Proton NMR - h1_latest
CDCl3, 399.78MHz, 21.1deg
5q
HO
Ph
H
O
EtOAc
f1 (ppm)

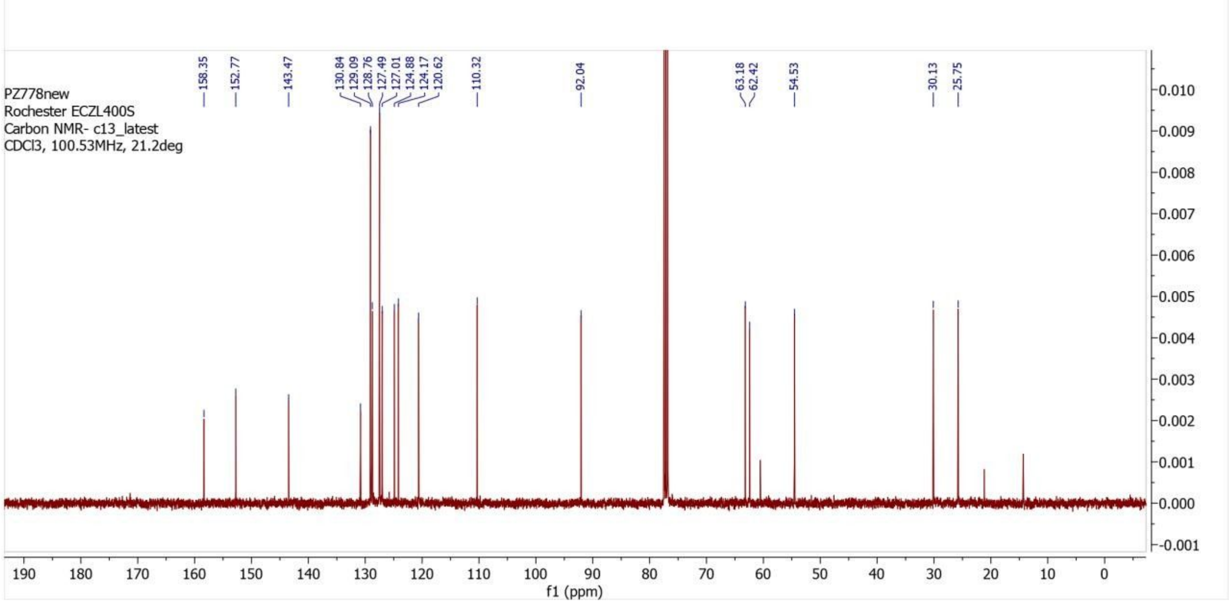
PZ778new
Rochester ECZL400S
Carbon NMR- c13_latest
CDCl3, 100.53MHz, 21.2deg
158.35
152.77
143.47
130.84
129.09
128.76
127.49
127.01
124.88
124.17
120.62
110.32
92.04
63.18
62.42
54.53
30.13
25.75
f1 (ppm)

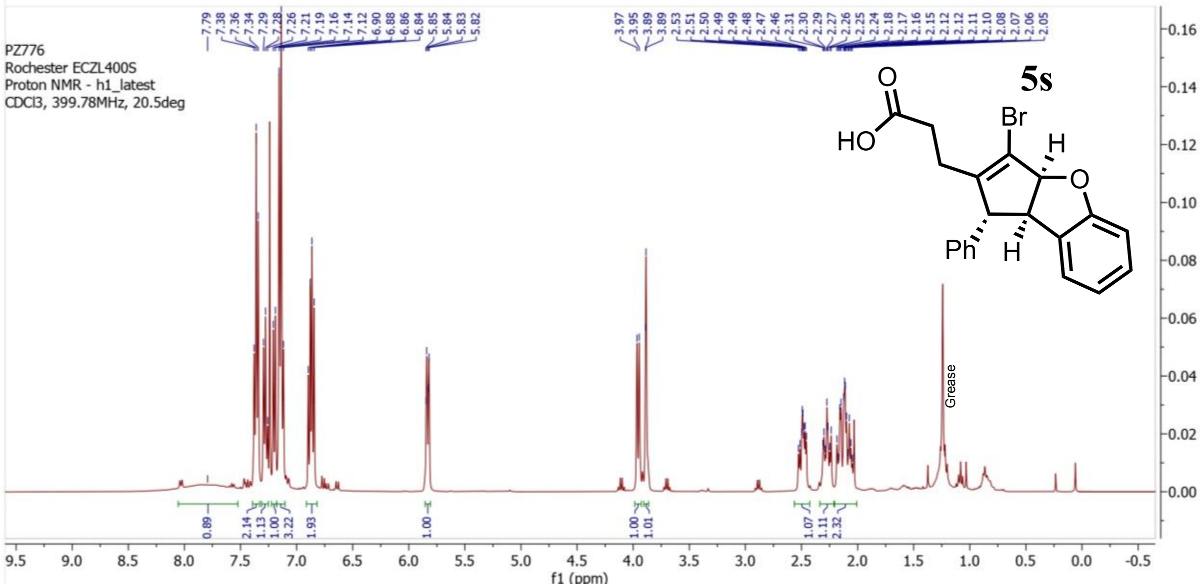
PZ776
Rochester ECZL400S
Proton NMR - h1_latest
CDCl3, 399.78MHz, 20.5deg
5s
Br
O
HO
H
Ph
Grease
f1 (ppm)

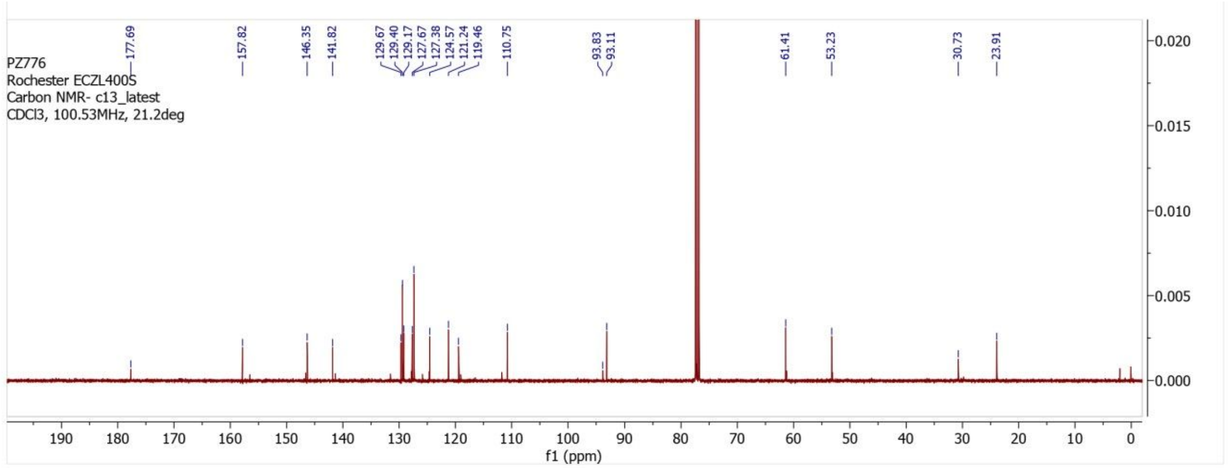
PZ776
Rochester ECZL400S
Carbon NMR- c13_latest
CDCl3, 100.53MHz, 21.2deg
177.69
157.82
146.35
141.82
129.67
129.40
129.17
127.67
127.38
124.57
121.24
119.46
110.75
93.83
93.11
61.41
53.23
30.73
23.91
f1 (ppm)

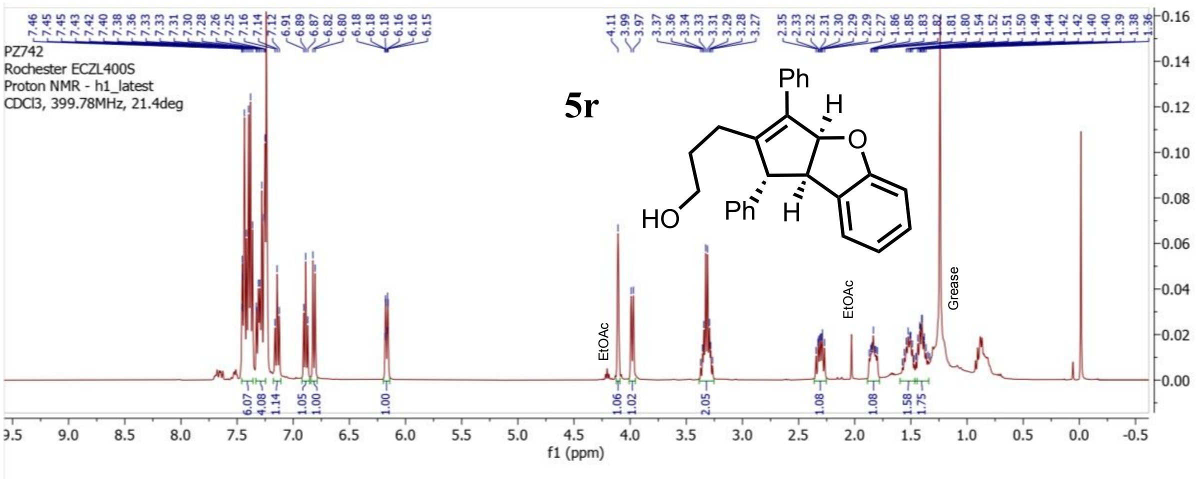
PZ742
Rochester ECZL400S
Proton NMR - h1_latest
CDCl3, 399.78MHz, 21.4deg
5r
Ph
HO
EtOAc
Grease
f1 (ppm)

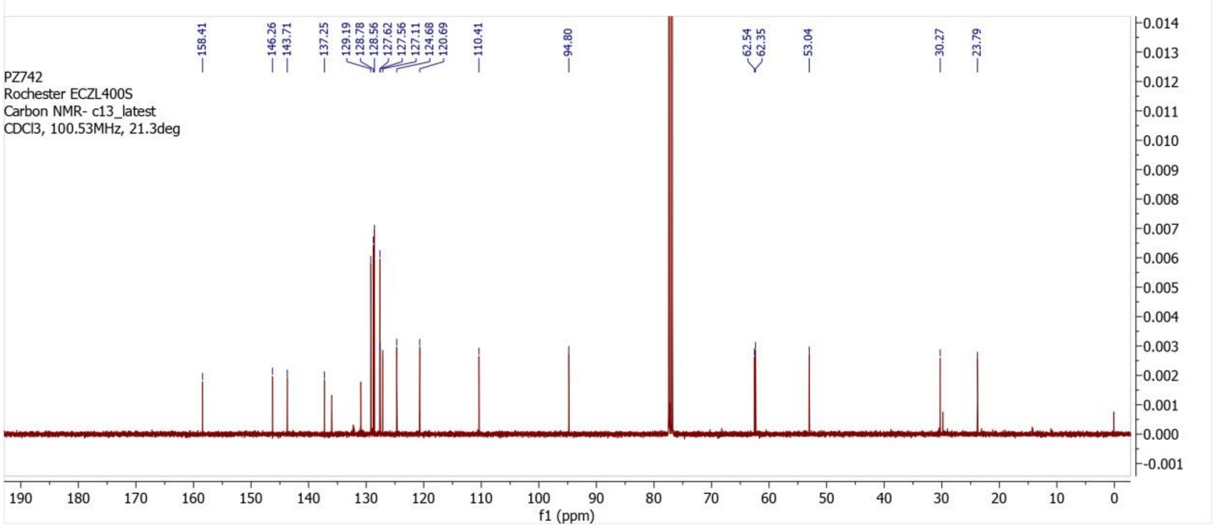
PZ742
Rochester ECZL400S
Carbon NMR- c13_latest
CDCl3, 100.53MHz, 21.3deg
f1 (ppm)
